# Network Topology and Interactomic Analysis Reveal the Regulatory Framework of the Humanin Protein Family (MTRNR2Lx Class)

**DOI:** 10.3390/biom16070981

**Published:** 2026-07-03

**Authors:** Mohd Shahzaib, Domenico Aprile, Gianluigi Laporta, Umberto Galderisi, Giovanni Colonna

**Affiliations:** 1 Department of Experimental Medicine, Biotechnology and Molecular Biology Section, Luigi Vanvitelli Campania University, 80138 Naples, Italy; mohd.shahzaib@unicampania.it (M.S.); lg24@outlook.it (G.L.); umberto.galderisi@unicampania.it (U.G.); 2 Department of Life Sciences, Health and Health Professions, Link Campus University, 00165 Rome, Italy; 3 Genome and Stem Cell Center (GENKÖK), Erciyes University, 38280 Kayseri, Turkey; 4 Medical Informatics Unit-AOU L. Vanvitelli, Università della Campania, 80138 Naples, Italy

**Keywords:** humanin, MTRNR2L, mitochondrial micropeptides, interactome, network topology

## Abstract

This study presents an in-depth analysis of an interactome comprising approximately 1033 nodes, focusing on its topology, reliability, and functional implications, with particular attention to the small mitochondrial proteins of the Humanin family and their nuclear-encoded MTRNR2Lx paralogs. The analysis, conducted through stringent high-reliability filters and experimentally supported interaction data, produced a curated network model in which approximately 70% of the retained interactions were supported by experimental evidence, providing a solid basis for network-based functional interpretation. The topology of the interactome showed scale-free and modular network characteristics, with hub and bottleneck nodes defining highly connected stress-response and regulatory modules. Humanin-related proteins, positioned at the periphery of the interactome, emerged as candidate modulatory nodes linking peripheral signaling interfaces to broader functional modules. Mitochondrial Humanin may contribute to early cytoprotective responses, including pathways associated with BAX-dependent apoptosis regulation, whereas nuclear MTRNR2Lx proteins appear to be connected to more sustained regulatory networks involving neuroprotection- and apoptosis-associated modules under chronic stress conditions. In particular, the MTRNR2Lx–FPR2/G-protein module, including GNB1, emerged as a candidate signaling interface that may contribute to the downstream organization of Humanin-related responses. This network-based distinction supports the view that Humanin-family peptides may operate as modulators of stress-response networks rather than as isolated effectors of intrinsic mitochondrial functions. Overall, the methodological approach, results, and proposed model provide new insights into the systems-level organization of Humanin biology and identify prioritized molecular candidates for future in vitro and in vivo validation in the context of neurodegeneration, apoptosis, and cellular stress.

## 1. Introduction

Human mitochondrial DNA encodes two major ribosomal RNA genes, *MTRNR1* (12S rRNA) and *MTRNR2* (16S rRNA), which are essential components of the mitochondrial ribosome and play a central role in mitochondrial protein synthesis [1]. While these genes primarily function in translation, accumulating evidence indicates that they also harbor short open reading frames that encode biologically active micropeptides [2,3]. In particular, a region within *MTRNR2* encodes Humanin (HN), a 24-amino-acid peptide originally identified for its neuroprotective and anti-apoptotic properties, especially in neurodegenerative disorders such as Alzheimer’s disease [4,5].

Researchers have identified a family of nuclear-encoded homologs, known as *MTRNR2*-like (*MTRNR2L*) genes, in the human genome, besides the mitochondrially encoded Humanin. These paralogous genes (*MTRNR2L1*, *L6*, *L7*, *L8*, *L10*, *L11*, and *L12*) encode peptides highly similar to Humanin. We collectively refer to these peptides as Humanin-like peptides [6,7]. Although scientists are still investigating their precise evolutionary origin, they believe these genes represent an adaptive expansion of mitochondrial-derived signaling molecules [8,9]. Importantly, nuclear *MTRNR2L* genes may provide a rapid and readily accessible source of transcripts, allowing cells to respond to stress conditions independently of mitochondrial peptide export [6,10]. Importantly, researchers detected Humanin peptides in the cytoplasm, which suggests that both mitochondrial and nuclear sources contribute to their functional pool [3,11].

Humanin and Humanin-like peptides play roles in numerous cellular processes, including cytoprotection, modulation of apoptosis, and regulation of the oxidative stress response [12,13]. Several studies have demonstrated their ability to interact with cell surface receptors, such as formyl peptide receptor 2 (FPR2), and to activate intracellular signaling pathways involving G proteins and downstream survival cascades [14,15,16,17,18]. However, despite these advances, the specific contribution of individual *MTRNR2L* genes to these biological effects remains poorly defined.

A major limitation in the field is the difficulty of distinguishing mitochondrial and nuclear Humanin isoforms due to their high sequence similarity and small size [8]. This technical challenge hampers the establishment of a clear genotype–phenotype relationship and limits our understanding of the specific roles of these variants in cellular physiology. Furthermore, researchers primarily focused on single peptides or pathways, and these investigations did not thoroughly examine the broader network of protein–protein interactions underlying Humanin peptide.

To overcome these limitations, a systems-level approach is required to investigate the molecular context in which Humanin peptides operate. In this study, we applied an interactomic strategy based on experimentally validated, high-confidence protein–protein interaction data to reconstruct the functional networks associated with Humanin and Humanin-like peptides [19,20]. This approach enables the identification of key regulatory nodes and interaction modules that reductionist analyses cannot capture.

Our results suggest that Humanin-like peptides encoded by *MTRNR2L* genes occupy a peripheral but functionally relevant position within the interaction network, suggesting a regulatory role in cellular signaling. In particular, we identified a core interaction module involving the FPR2 receptor and associated G proteins, which likely mediates the downstream effects of these peptides. These findings provide a model for new insights into the molecular mechanisms underlying Humanin function, supporting a role as context-dependent regulators of cellular homeostasis and highlighting the importance of network-based approaches for the study of small bioactive peptides.

## 2. Materials and Methods

### 2.1. Rationale

Interactomic networks are essential tools for modeling biological and pathological processes [21]. However, large-scale datasets often include heterogeneous interaction types, particularly those derived from text-mining or computational predictions, which may not accurately reflect physiological interactions [22].

Current literature lacks detailed in vivo information regarding the temporal sequence and contextual conditions under which protein–protein interactions occur [23]. Although large-scale omics analyses predict thousands of interactions, researchers have experimentally validated and functionally characterized only a limited fraction [20,24,25,26]. Estimates suggest direct experimental evidence supports that fewer than 20% of predicted interactions, highlighting the need for stringent data selection to ensure biological relevance.

To address this limitation, we adopted a systems biology-oriented approach focused on reducing network complexity while preserving high-confidence interactions. Specifically, we used experimentally validated low-throughput (LT) interactions, confirmed in vivo by at least two independent methods, as functional seeds. We retrieved these interactions from curated databases, including BioGRID, IntAct, and MINT, which store manually annotated physical interaction data [27,28]. Subsequently, we enriched the networks and progressively filtered them based on topological criteria, removing approximately 90% of low-confidence connections. This strategy enables the identification of biologically meaningful gene networks and supports the inference of phenotype-specific interaction patterns.

### 2.2. BioGRID

BioGRID (Version 4.4.235, released 1 July 2024; https://thebiogrid.org/) (accessed on 5 July 2025) is a curated biomedical database that collects protein and genetic interactions derived exclusively from experimental studies performed in living systems [27]. The database integrates both low- and high-throughput experimental data through a standardized curation process. Curators manually review and annotate each interaction, ensuring high data quality and reliability. For this study, BioGRID served as a primary source of experimentally validated protein–protein interactions.

### 2.3. STRING: Database and Network Construction

STRING (Search Tool for the Retrieval of Interacting Genes/Proteins; Version 12.0, https://string-db.org/, released on 26 July 2023) (accessed on 1 November 2025) is a database that integrates both known and predicted protein–protein interactions. These include direct (physical) and indirect (functional) associations derived from experimental data, computational predictions, and curated pathway databases [28,29].

In this study, we used STRING to expand the interaction network by systematically incorporating both first-order (direct) and second-order (indirect) interaction partners, up to 500 of each. STRING assigns confidence scores to each interaction based on multiple evidence channels, including experimental data, curated databases, co-expression, and computational predictions. These scores are calibrated against known pathways (e.g., KEGG) and combined into a single probabilistic confidence score ranging from 0 to 1. To ensure high reliability, we applied dynamic thresholding with confidence cutoffs between 0.700 and 0.999. The optimized network achieved approximately 70.2% high-confidence interactions; the remaining 29.8% measured residual uncertainty. Isolating a single data source for experimentally validated interactions improves the interpretability of interaction networks, providing a much more reliable structural and functional context that more closely reflects biological reality.

### 2.4. Protein Enrichment Strategy

Enrichment was conducted (29 December 2025) in two clearly defined phases to balance exploratory breadth with methodological rigor. In the first phase, we applied a fully permissive strategy with a confidence score of 0.15, activating all available interaction channels and setting the enrichment to include 500 first-order and 500 second-order proteins. This initial configuration was intentionally broad, maximizing the retrieval of potentially relevant interactions and providing the most comprehensive overview of the molecular landscape associated with the humanin study area. In the second phase, to reduce the risk of bias introduced by annotation-driven enrichment and to obtain a more controlled and interpretable network structure, we adopted a more conservative strategy. Specifically, we first uploaded the complete set of experimentally validated proteins to STRING and disabled both first- and second-order interaction expansion to prevent automatic node addition. Starting from this curated core, we progressively introduced interaction partners according to predefined confidence thresholds, allowing the network to expand in a stepwise and transparent manner. First-order interactions were added first to capture the most direct functional associations. Second-order interactions were included only when necessary to improve network connectivity or to reveal potentially relevant indirect relationships. Throughout this process, a high-confidence threshold (score ≥ 0.7) proved to be the most effective setting, as it consistently maintained biological relevance while limiting the inclusion of weak or potentially false-positive associations.

### 2.5. Cytoscape and Network Topology Analysis

We performed network visualization and topological analysis using Cytoscape (version 3.10.1, released on 31 August 2023) and the Network Analyzer plugin (accessed and conducted on 1 February 2026) [30,31], representing protein–protein interaction networks (PPINs) as graphs. Nodes correspond to proteins, and edges represent interactions. Several topological parameters, such as network density, clustering coefficient, centralization, and network diameter, were evaluated.

Node degree (k), defined as the number of connections per node, was used to characterize network structure. To assess scale-free properties, we analyzed the degree distribution P(k) and fit it with a power law. Calculating the coefficient of determination (R^2^) allowed us to evaluate the goodness of fit. Using centrality measures allowed us to identify key nodes with high topological relevance. CentiScaPe was used to compute centrality metrics, including degree, betweenness, and closeness centrality, enabling the identification of hub nodes and functionally relevant proteins within the network [32].

### 2.6. HUB-and-Spoke Model Evaluation

To characterize the network topology, we evaluated scale-free properties using the degree distribution exponent (γ) [33]. We estimated the expected maximum node degree (natural cutoff) using the following relationship: k_max_ ~ k_min_ N^(1/(γ−1))^, where k_max_ and k_min_ represent the maximum and minimum node degrees, respectively, and N is the total number of nodes in the network [33,34,35]. This analysis provides insights into hub structure and network robustness.

### 2.7. Clustering Analysis

We performed cluster analysis using the K-means algorithm, an unsupervised centroid-based method implemented in the STRING framework(accessed on 25 February 2025) [36]. It is an effective iterative partitioning algorithm, which determines the number of clusters based on their centroids, ensuring high within-cluster similarity and low between-cluster similarity. The value of K, the number of clusters, is one of the main drawbacks of this algorithm. We tested various values. The number of clusters (K = 5) was selected empirically after testing multiple configurations, optimizing for cluster compactness, functional coherence, and statistical significance. As a result, proteins were grouped into functional clusters based on network connectivity and biological relevance.

## 3. Results and Discussion

### 3.1. Optimization of Interactome Calculation Procedures

Human micropeptides and their nuclear-encoded paralogs are tiny mitochondrial proteins distributed throughout the cell and extracellular space [37], but the molecular mechanisms underlying their activity are poorly understood [38]. Interactomics is a systems biology approach for studying molecular interaction networks to explore causal mechanisms at a deep metabolic level [39,40]. Its ability to provide reliable predictive models depends on the analysis of PPIs. The more accurate these interactions are, the better the prediction, as supported by solid experimental validation [22,23,41]. Otherwise, we risk producing hypothetical and unrealistic models whose behaviors are disconnected from empirical evidence. These conditions require careful selection of interactions to analyze, as well as precise filtering of interactions computed in the interactome.

We relied on specialized databases reporting physical interactions validated by biophysical and/or biochemical experiments conducted in vivo or in vitro. We selected proteins that interact with small humanin-class proteins, focusing only on those exhibiting LT interactions. Therefore, all the features highlighted in this study are based on data validated by at least two experimental methods. Table 1 presents the 42 selected Humanin/Humanin-like seed interactors. Detailed supporting evidence, including original references, experimental methods, evidence type, and in vivo/in vitro classification, is provided in Supplementary Dataset S1.

This protein set served as a functional seed in STRING, a leading interactomics platform with tools to assess interactomic properties. We expanded the initial set by adding 500 first-order and 500 second-order proteins, using a confidence score of 0.150 and all seven source channels. Figure S1 shows the resulting interactome, which contains 1041 nodes and 267,931 edges. This unconventional, low-confidence approach, based on about 10,000 PubMed publications, maximized information extraction from the human proteome and outlined current knowledge on humanins, whether accurate or not (Figures S2 and S3, Table S1). This broad view is necessary to define the functional landscape explored here. More rigorous approaches that rely on high-quality interactions (>0.700) will help identify and filter out unreliable information. Filtering reduces the number of interactions while emphasizing potentially biological ones [33]. Thus, changes in average node degree indicate how many functional relationships each node has and with which partners. A node’s connectivity depends on network architecture (how elements are organized and linked) and topology (the pattern and distribution of connections). Biological networks typically show a scale-free degree distribution with small-world properties [33,42]. Here, the average node degree is exceptionally high, at 514 interactions per node. The high number of high-degree nodes differs from the lower values typically found in biological networks, distorting the degree distribution, creating connection imbalances, and affecting interactome behavior (see Supplementary Dataset S2 for specifics). This suggests that numerous papers rely on unvalidated hypotheses or questionable assumptions about mitochondrial peptides. Such a trend fosters speculative epistemic bubbles, in which unproven claims are treated as facts. Therefore, reliable functional interpretation requires rigorous experimental validation. In the humanin literature, this apparent hyper-functionality assigns nodes roles beyond their true functions, leading to misinterpretation [41].

**Figure 1:**
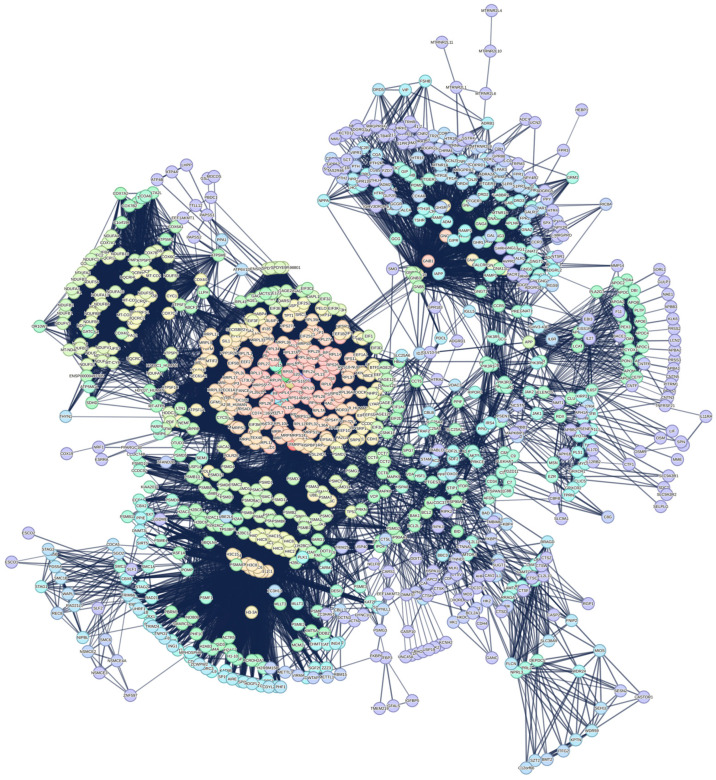
Interactome-1033 was obtained from the initial 1041-node interactome after removal of eight unconnected nodes. The remaining 1033 nodes were used to reconstruct the network using the same settings: confidence score ≥ 0.700 and exclusion of the text-mining channel. Experimentally supported interactions accounted for 69.23% of the network, as reported in Supplementary Dataset S3. The network shows a compact central core and multiple peripheral functional modules. The main topological parameters are provided in Supplementary Dataset S4.

Figure S4 (Table S2) shows the interactome from Figure S1 recalculated with a confidence score of 0.700 and without the text-mining channel. The resulting network is more dispersed, with a central core, several peripheral modules, and some unconnected nodes. Table S3, and Figures S5 and S6, show that it has lost thousands of functional terms and that the average node degree has dropped to 51.9. Although stringent filtering removed less significant terms, the interactome can be further refined. Figure 1 shows the network from Figure S4 after pruning eight unconnected nodes under the same settings. The revised interactome contains 1033 interacting nodes, referred to as Interactome-1033.

We observe that MTRNR2LX proteins are at the periphery of the interactome, marking a substantial shift in interpretation. If they were central regulators of mitochondrial metabolism, they would cluster within the network core and connect strongly to mitochondrial enzymatic pathways. Instead, their peripheral placement suggests an interface-like role [43,44,45]. This topological interpretation is supported by their high eccentricity values, which indicate a large distance from the network’s center of mass (Supplementary Dataset S4).

This finding suggests that the network does not identify humanins as peptides with direct mitochondrial functions. Rather, their effects appear to be mediated through non-mitochondrial proteins, consistent with a more external, cytoplasmic role, likely involving cytosolic or nuclear humanins. If, as shown later, the enriched network modules are associated with apoptosis and neuroprotection, this cytoplasmic localization would influence those processes. Importantly, this role is not inherently neuroprotective; rather, it involves modulating pathways that govern apoptosis and neuronal stress. These effects can be understood as emergent properties of network topology [46], arising from interactome modules rather than from the isolated activity of individual peptides. Thus, the cellular functions attributed to humanins derive from specific interactions among functional modules that place them at the network edge. This challenges the common view that humanins should primarily be interpreted through a mitochondrial framework. What emerges from the interactome is instead a role for these small proteins in regulating apoptosis or neuroprotection within cytosolic and nuclear modules.

From a graph-theory perspective, peripheral nodes are important for system plasticity [43,44,45]. If MTRNR2LXs occupy the interactome’s edge, they may provide exogenous or endogenous inputs to stable central modules. Their role in the system, therefore, depends on multiple, complex interactions with specific nodes, and cellular functions may depend on the combination of these distinct connections.

A comparison between the confidence-score distributions of the initial interactome-1041 (Figure S1) and the final interactome-1033 (Figure 1) is also informative (see Supplementary Dataset S3 for full details). Each final PPI score represents the estimated probability that a data channel contains a true protein–protein association. In the experimentally determined interaction channel, in vivo-validated interactions are reported on a 0–1 scale, with values greater than 0.700 indicating stronger statistical confidence and biological reliability. Table 2 quantitatively compares these confidence scores for experimentally validated protein–protein interactions (PPIs) between the two interactomes.

The table shows that filtering interactions by confidence level profoundly changes the biological interpretation. In interactome-1041, protein–protein interactions (PPIs) with scores between 0.700 and 0.999 represent only 0.74% of the total, meaning that 99.03% of the 267,231 interactions are hypotheses that have not been experimentally validated. By contrast, in interactome-1033, 69.23% of interactions have been experimentally validated in vivo within the same confidence range. Although these estimates may be approximate, the reliability gap between the two interactomes is highly significant and rarely seen in interactomic analyses. Thus, interactome-1033 is enriched in reliable, validated interactions, so confidence in the biological functions it expresses should be very high. An interactome is a molecular map of a specific phenotypic state, showing which proteins interact with one another [20,47]. If the information is accurate, this map can infer functions, regulatory relationships, and even pathologies. In this context, interactome-1033 captures more stable forms of functional differentiation, with a topology that reflects the real boundaries and limits of the interactions underlying its stability and function. Then, an interactome is not merely a static interaction map but a dynamic system that stabilizes and regulates cellular functions through nodes defining their properties and boundaries. It also helps explain how molecules or cells specialize in specific functions. These nodes are crucial because they determine which molecules can connect and how, and such boundaries are essential for system stability and proper function. Comparing Tables S1 and S3 shows that interactome-1033 has lost 71.92% of the primitive-enriched functional terms, especially in GO Biological Processes, while the others declined proportionally. This offers an indirect but accurate measure of how much information in this field may be incorrect, with untested hypotheses mistakenly treated as scientific truth.

### 3.2. Principal Characteristics of the Interactome-1033

We transferred interactome-1033 to Cytoscape [31] and analyzed it using Analyze Network [32]. This analysis generated quantitative values for many topological parameters. This enabled the evaluation of the interactome’s topological features. Table 3 presents statistics for these topological parameters.

The network diameter, the greatest distance between the two most distant nodes, is 7 edges (hops). This relatively low value suggests an efficient, fault-tolerant network in which nodes remain close even during disruptions [48]. It also ensures connectivity, since any pair of nodes can be linked by a path of at most 7 hops. Biologically, this means a signaling packet never passes through over 7 intermediate nodes. The radius is 4, the minimum eccentricity in the network, meaning the most central node lies 4 hops from the most peripheral nodes [49]. Together, a diameter of 7 and a radius of 4 suggest an elongated structure with arm-like extensions: the network has a center, but its lengthwise span is typical of non-symmetric systems with widely separated peripheral nodes. Examination of the interactome supports this interpretation. The characteristic path length, which complements diameter by measuring the average shortest path length, is 2.91, indicating that interactome-1033 exhibits typical small-world properties [50]. Although some node pairs are 7 hops apart, the network remains well-connected overall, with an average path length of fewer than 3 hops. The density is 0.056, showing that the network is very sparse: only 5.6% of all possible connections are present [51]. While diameter and path length describe how far or how quickly communication travels, density reflects how tightly connections are packed. In a network with such low density, the few bridges available often cause detours via intermediate nodes, which consequently increases both the diameter and the path length. Network heterogeneity (1.127) and centralization (0.233) describe how connections are distributed and how strongly central nodes influence the system [52]. High centralization often reduces diameter, but, like path length, it does not reflect physical distance. Overall, the combination of very low density (0.056) and a low average path length (2.91) is characteristic of an efficient small-world network. Despite having few edges, their arrangement keeps nodes close. However, a diameter of 7 shows that the periphery, or critical path, is about twice the average path length. If density dropped further, diameter and path length would rise sharply as routes became more convoluted or the network fragmented. Thus, network density reflects structural constraints and interaction coordination requirements (topological cost), while distance reflects communication capability. In summary, this network combines high throughput (average path length of 2.91) with very low cost (density of 5.6%). These parameters suggest a core–periphery, or hub-and-spoke, structure in which the network serves as a bridge between the core and the periphery, enhancing efficiency. Below, we present the details that support this interpretation.

The interactome-1033 network enables rapid communication between peripheral modules and central control nodes, supporting feedback and systemic regulation. Its short characteristic path length (2.91) indicates that peripheral signals can reach the core in fewer than three steps, so the periphery is not isolated. At the same time, the larger diameter (7) indicates a hierarchical structure in which most nodes cluster near the center while a few remain at the edges, allowing peripheral modules to act as sensors or regulators without dense connectivity.

The moderate centralization value (0.233) suggests identifiable core nodes but not an overly centralized structure, supporting two-way influence between the center and the periphery. This architecture combines efficiency and vulnerability: low density and short distances enable fast, energy-efficient communication, but they also make the network dependent on a few critical nodes through which effects can spread quickly. In addition, the high local clustering coefficient (0.761), despite the low density (0.056), indicates that the network is highly modular, with interactions concentrated in cohesive local groups or functional cliques.

In summary, our filtering protocol optimized the interactome-1033 structure and enabled its peripheral modules to communicate rapidly with control centers, supporting feedback and systemic regulation. We also observe a high average local clustering coefficient of 0.761 despite the low density of 0.056, adding an important dimension to this highly specific network model. This suggests the network is not only efficient but also highly modular: although it has few total interactions, those interactions are concentrated in closed local groups. If node A connects to B and C, B, and C are also likely to connect, indicating that the network comprises highly cohesive cliques, or groups of functional units.

### 3.3. Interactomic Parameters Fit the Small-World Model

Let us consider our data under the “Small-World” model [33,53]. It shows all key Watts–Strogatz features [54]: a high clustering coefficient (0.761), indicating strong local specialization, with edge nodes collaborating in dense modules; a low path length (2.91), meaning few “hops” are needed between modules; and a diameter of 7, allowing longer paths to connect opposite ends of the structure.

In this context, the periphery’s ability to modulate the core takes on a new dimension: local processing [55]. Edge modules intensively process information due to their high clustering. Once an edge module “decides” something, it sends the information to the core through very few long-distance links or shortcuts rather than scattering it randomly. This architecture suits systems that need both local autonomy and global responsiveness.

A high clustering coefficient protects modules: a small failure in one node does not destroy the module because neighboring nodes compensate, ensuring robustness to noise [56,57]. However, because the overall density is low (0.056), links between dense clusters and the core depend on a few connector nodes, often called bottlenecks or bridges. If these nodes fail, entire modules can become isolated [33,45,58].

In conclusion, interactome-1033 is strongly organized into districts or modules. Each district is nearly a closed group (clustering coefficient 0.76), yet the system ensures each is within 3 steps of the core. This resembles neural circuits, agile enterprise organizations, and some critical infrastructures.

### 3.4. The Tiny Mitochondrial Proteins Are Peripheral: What Could It Mean?

**Figure 2:**
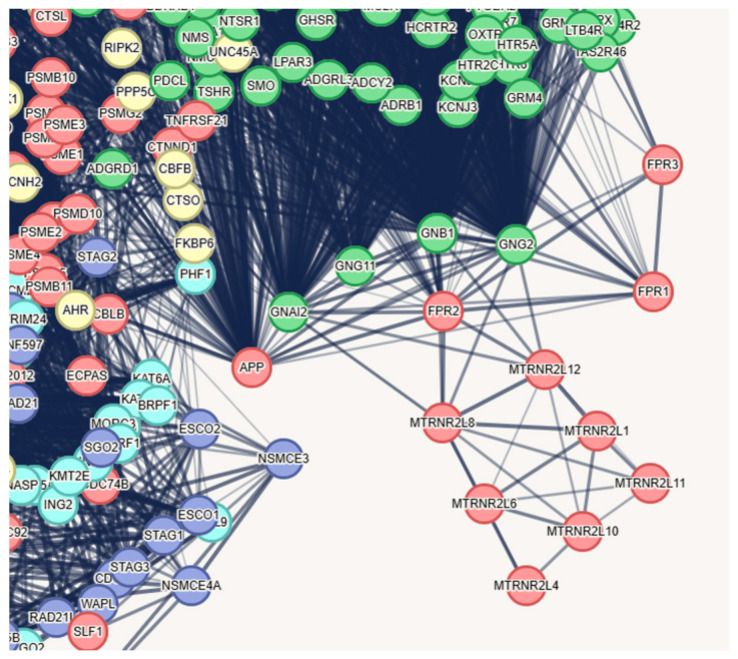
Peripheral location of the humanin nuclear cluster. Close-up view of the peripheral network region containing MTRNR2L family members and their associated interactors. MTRNR2L8 and MTRNR2L12 are connected with G-protein-related nodes, including GNG2, GNB1, GNAI2, and FPR2, suggesting a candidate interface between Humanin-like peptides and GPCR-associated signaling modules.

As shown below, the topological parameters of MTRNR2LXs support this placement [44,45]. Here, we examine the interactome context. Across interactomes, these small proteins consistently occupy peripheral positions, and interactome-1033 follows the same pattern. Figure 2 zooms in on this peripheral zone and shows how these proteins relate to subgraph nodes.

The humanin group is linked to functional module proteins by MTRNR2L8 and MTRNR2L12, which act as key mediators. They interact directly with GNG2, GNB1, GNAI2, and FPR2, which form a tightly connected cluster linked to the underlying functional module. GNG2, GNB1, and GNAI2 are guanine nucleotide-binding proteins (G proteins) that act as molecular switches in intracellular signaling, often downstream of cell-surface receptors. They cycle between inactive and active states and, when activated, transmit signals by interacting with other molecules. FPR1, FPR2, and FPR3 belong to the formyl peptide receptor (FPR) family, a group of G-protein-coupled receptors involved in immune defense and inflammation. They detect N-formylated peptides, which damaged-cell mitochondria also release as danger signals. FPR2 stands out for its broad ligand binding, recognizing both endogenous and exogenous ligands, formylated and non-formylated. Depending on the ligand and context, it can drive pro-inflammatory, anti-inflammatory, or inflammation-resolving responses. FPR2 interacts closely with G proteins, especially the GNAI2, GNB1, and GNG2 subunits. Our interactome-1033 data and external databases (STRING and BioGRID) suggest that MTRNR2L8 and MTRNR2L12 have more complex associations than previously reported. The data show physical interactions among themselves and with G-protein components, supported by experimentally validated, high-confidence evidence (0.800–0.999). These results suggest that MTRNR2LX proteins influence signaling through more complex, less direct mechanisms, including physical contact with G proteins, which may shape downstream functional fluxes and cellular responses. However, assessing the interaction between humanin and FPR2 first requires confirming that the interaction between unformylated MTRNR2L8/12 and FPR2 is physically possible. This assessment is provided in the Supplements (p. 4). In conclusion, although this view differs from the current literature, it is supported by robust evidence.

### 3.5. Characteristics and Properties of the Interactome as a Population

**Figure 3:**
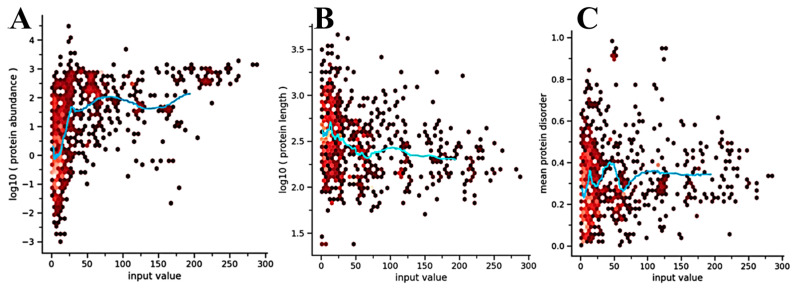
Characteristics and properties of the population of molecules that populate the interactome-1033. STRING performed these calculations across the entire population of molecules that comprise the interactome. Statistics: (**A**) Pearson’s r value: 0.488, Pearson’s *p*-value: 1.00 × 10^−32^, and BP-R2: 0.454. Data from the PaxDb: Protein Abundance Database (https://pax-db.org/) (accessed on 28 February 2025). (**B**) Pearson’s r value: 0.126, Pearson’s *p*-value: 0.00011. (**C**) Pearson’s r value: −0.29, Pearson’s *p*-value: 2.13 × 10^−19^, BP-R2: 0.199.

Figure 3 emphasizes the main characteristics of the molecular population within interactome-1033. The Pearson r value of 0.488 (Figure 3A) shows a moderate positive correlation between seed-protein interactions and the proteome, increasing some protein groups while reducing others and their associated functions [59]. The graph also shows many proteins with anomalous abundance values: many exceed +1, whereas fewer fall below −1. This pattern supports the idea that seed proteins reshape functional protein groups within the proteome. The BP-R2 value of 0.454 indicates that the regression model predicts the data well and suggests that regulation, though incomplete, strongly influences final protein concentrations and phenotypic responses beyond genetic input alone [59]. We must also consider how the crowded cellular environment of interactome-1033 shapes these population features. In addition, Figure 3B,C show many long intrinsically disordered proteins (IDPs), reinforcing the network parameters discussed above.

#### 3.5.1. Network Implications of Crowding and IDPs

High protein abundance and a large fraction of intrinsically disordered segments or proteins suggest a dynamic, adaptive network built for rapid regulation rather than rigid, static interactions [60]. The combination of low network density (≈0.056) and high local clustering (≈0.76) indicates a small-world network that supports both local specialization and fast global communication through strategic bridges (path length ≈ 2.91; diameter 7). This organization likely forms autonomous modules that transmit information quickly across the system, much like neural circuits or complex metabolic networks [61,62]. Cellular crowding also drives short-range interactions: nearby molecules connect more easily than molecules that must diffuse to distant partners. Consequently, the network develops concentrated local groupings, even though its overall structure is sparse, causing nodes to unite into closely knit communities or clusters.

#### 3.5.2. Role of Flexible Nodes and Short Distances

The protein population contains many intrinsically disordered proteins (IDPs), which make up approximately 50%. These flexible proteins lack a stable three-dimensional structure until they bind a partner [63,64]. Because they have many binding sites, they interact with multiple partners and act as multifunctional interactors [64,65,66]. IDPs often serve as highly connected central nodes [67]. Their flexibility lets them bind many partners sequentially or simultaneously and create bridges or shortcuts, which helps explain the low average path length (≈2.91) despite the network’s low density. In this way, IDPs act as highly efficient switchboards that rapidly connect clusters. They may also promote hub formation by interacting extensively within protein complexes or functional modules. Under stress, IDPs help Humanins connect quickly with multiple partners.

#### 3.5.3. Rapid Modulation and Fluctuations in Population Abundance

Flexibility appears to define the core structural feature of the interactome-1033 population rather than a dynamic variable linked to expression [68], given the weak correlation between mean protein disorder and input values (r = 0.126). Instead, the system regulates itself through large shifts in abundance (protein excess or deficiency, >1 or <−1). Rather than changing the conformation of flexible proteins, it adjusts their abundance to modulate the interactome [69,70]. Overall, these data portray an interactome that balances efficiency with specialization. Flexible IDP nodes use a crowded environment to build local clubs and global bridges, while quantitative changes in these components regulate the system [68,69,70]. Later quantitative topological analyses will test these predictions.

### 3.6. Power Law of Interactome-1033

**Figure 4:**
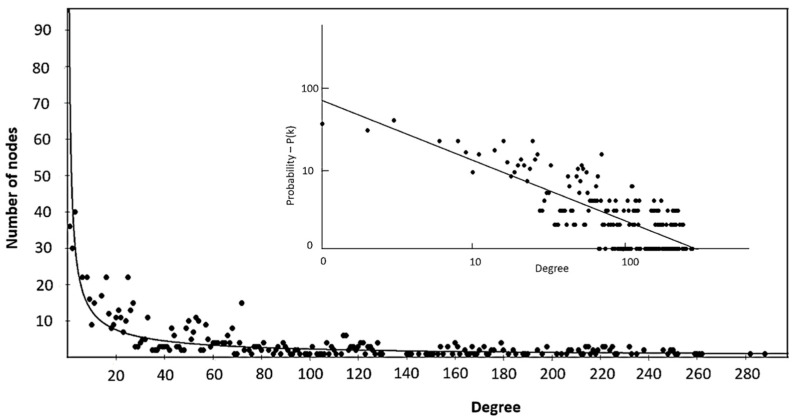
Node Distribution of interactome-1033. The distribution follows a power-law scaling. In the inset, we present the same nodes on a log–log scale, along with the best-fit line (f(x) = ax^−0.74^; R^2^ = 0.556). The slope is −0.392, calculated from the best-fit line in the log–log inset.

Figure 4 shows the power-law distribution of node degrees [34,71]. Visual inspection of the graph, especially the log–log inset, indicates that the data points deviate markedly from the regression line, particularly in the high-connectivity region on the far right. Several points lie well above the trend line, corresponding to nodes with degrees higher than predicted by the theoretical model *f*(*x*) = *ax*^*b*^.

Given the rigorous filtering applied to obtain these data (69% of interactome interactions have a confidence score > 0.700; see Supplementary Dataset S3), the observed deviations are likely genuine biological signals rather than methodological artifacts. The interactome contains 1033 nodes, about 10% of which are the most connected hubs (degree ≥ 160), forming the long-tail population. This long tail indicates a highly centralized network and supports the idea that the graph is scale-free [71,72,73]. However, evaluating the fit together with the topological parameters already analyzed provides a clearer picture. In the power function *f*(*x*) = *ax*^*b*^, parameter b is the power-law exponent that defines network topology. Here, gamma (the absolute value of the exponent) is 0.74. In real biological networks, gamma usually ranges from 2 to 3, so *b* = −0.74 is unusually low. A gamma <1 is mathematically extreme for an interactome, implying that the average degree diverges much faster than in a standard network. Such a low value indicates a heavy tail in which a few hubs exert disproportionate control. Under this winner-takes-all dynamic [74], these nodes receive most connections, suggesting a system dominated by multi-protein complexes that monopolize interactions rather than a simple hierarchy.

In this context, even a flat slope suggests a highly centralized network [75], which is unusual for a standard protein interactome, typically more hierarchical. Thus, deviation from the Barabási–Albert model would indicate not a different topology, but a hyper-centralized architecture [76]. The interactome is therefore less a democracy of proteins than a rigid hierarchy optimized to channel peripheral signals toward specific critical decision-making centers.

The correlation coefficient (R^2^ = 0.556) is also low. Systems biologists usually expect R^2^ values above 0.80 or 0.90 to classify a network as scale-free. Thus, R^2^ = 0.556 means the model explains only 55.6% of the variance, suggesting either a different degree-distribution pattern or substantial network noise. Although the *p*-value from the interaction analysis (1.0 × 10^−16^) is highly significant, it only shows that the correlation is not random and does not imply a good fit to the standard model. While high fits (R^2^ > 0.8) are common in networks based on computational predictions, our lower value likely reflects the rigorous filtering applied, with 69.2% of interactions experimentally validated in vivo [34,36,77]. A topologically noisy but biologically authentic model is more informative than a theoretically perfect but speculative one. Models with R^2^ > 0.9 in the literature often include predicted and heavily studied interactions, yielding aesthetically perfect but biologically less reliable graphs.

The local clustering coefficient (0.761) offers a clearer picture, indicating a preferential-attachment dynamic, although some technical differences must be considered [78,79]. In a purely scale-free network, clustering is usually low; therefore, this high value, together with an imperfect power law, suggests a modular-hierarchical structure. This deviation from the standard model reflects modularity, and the departure from the straight line in the high-degree region indicates that hubs attract more than expected.

However, a high clustering coefficient (0.761) in a noisy power-law network indicates a scale-free, hierarchical-modular structure. This value shows that a node’s neighbors are strongly interconnected, forming dense modules or “cliques” [80,81]. It suggests that some nodes have not only acquired new ties but also become aggregation centers where information or interactions are highly redundant. Thus, the weak power-law fit is counterbalanced by strong local clustering. Overall, the 1033 interactome appears not as a uniform network but as highly specialized cliques linked by dominant hubs. Because the R^2^ index is low (0.556) and 30.77% of interactions (see Supplementary Dataset S3) lack experimental validation, noise appears in the distribution’s tail, especially for degrees from 50 to 288, where nodes progressively shift toward higher degrees. Therefore, nodes with degrees above those predicted by the model (*b* = −0.74) dominate growth. In a protein interactome, these may represent key multi-protein complexes or “promiscuous” proteins that interact with various partners [66,67]. The substantial proportion of IDPs (approximately 50%) in the interactome is therefore notable. The low R^2^ captures the transition between local module specialization and global centralization. Similar distributions occur in biological systems under strong evolutionary pressure or in highly specific phenotypic states, where rapid response outweighs statistical regularity [71,82,83].

The node power-law properties may also be explained at the molecular level. A common feature of these nodes is an intrinsic property called fitness [72,73], which promotes preferential attachment and increases connectivity [74,75]. Fitness depends on each node’s structural and/or functional properties. In protein–protein interactions, structural disorder in interface motifs often mediates transient interactions, generating multistate complexes with dynamic subunit clustering. Intrinsic disorder is therefore a key feature of transient interactions, which promote entropy-driven conformational states [76,77,78]. These interactions impress the behavior of the most connected nodes [79], underscoring the role of intrinsic structural disorder in protein–protein networks that underlie and regulate node interactions.

Barabási proposed the Bianconi–Barabási, or Fitness, model to explain how nodes with different properties acquire connections at different rates [72,73]. In this model, a node’s growth rate depends on its fitness, determined by intrinsic protein properties such as secondary structure, solubility, binding affinity, flexibility, and functional specificity [84,85,86,87]. This provides a physical basis for the formation of dense, functionally connected subgraphs with highly connected central nodes, typical of protein networks. The model predicts a fit-get-richer dynamic in which node degree depends on fitness, enabling new links both between new and existing nodes. In real systems, this dynamic alters connectivity and can remove nodes that lose interactions. Intrinsically disordered proteins share many of these features, and we also assessed the prominence of disorder in the interactome. This helped clarify both the physical basis of subgraph formation and the network’s topological evolution [88], as disorder plays a key role in interactions. In complex networks with various subgraphs, central high-degree nodes connect to lower-degree nodes within subgraphs, preventing the network from forming a single large component.

The emerging picture is that hubs interact extensively within protein complexes or functional modules. They show high local assortativity, the so-called “Rich-Club Effect” [89]. Thus, although the network is globally disassortative (hubs do not connect randomly across it), it is locally assortative, forming very dense functional nuclei [90]. This explains the high clustering coefficient (0.761). These networks, therefore, show a hub-and-spoke organization [91,92,93]. The globally disassortative distribution, highlighted on the logarithmic scale, suggests that the network is intrinsically difficult to represent as a single large component. The negative slope of the log–log plot confirms that higher-ranked nodes are less likely to interact with one another [33,76]. Yet the high clustering value (0.761) also suggests that these central nodes are “alone” because they lie at the core of densely interconnected modules. Thus, a “rich get richer” dynamic is plausible, but it seems to operate at the sector level. Rather than a single “winner takes all,” several “winners” have formed their own high-density “neighborhoods” (modules). In this scenario, high-degree nodes are the most attractive, and their acquisition rate exceeds the network’s growth in node number of nodes. Within each module, the dynamic becomes “winner takes all” [94]. This feature often reflects the topological organization of central nodes in the “Hub-and-Spoke” model, where all nodes are a short distance from one another [95], and, as we will see later, is also a specific feature of our interactome.

In summary, the network is not “standard” but highly modular and hierarchical, with central modules comprising either giant molecular complexes or compact functional modules. The low R^2^ (0.556) is not a flaw but mathematical evidence that the network is neither randomly distributed nor purely scale-free; instead, it is driven toward these macro-modules. The exponent −0.74 reflects the functional “fusion” among these hubs, while the flat slope reflects the overrepresentation of stable complexes (as we will see later, ribosome/proteasome) that act as major connection attractors. Indeed, the first 100 hubs (see Supplementary Dataset S2) are all ribosomal proteins. The physical basis of this pattern also lies in the fact that approximately 50% of proteins in the interactome are disordered proteins (IDPs), which can act as “bridges” (bottlenecks) between these large complexes and the network periphery. This structure, in which a few “winners” (the hubs of large complexes) dominate their “neighborhoods” (modules), is the signature of a biological system optimized for rapid, specific responses rather than statistical regularity. The power-law therefore does not mark a departure from the Barabási–Albert model, but shows that the interactome has evolved a rigid, highly efficient hierarchy in which the periphery “feels” and the core “executes” almost instantaneously. This analysis, based on mathematical and topological principles and without introducing explanations or functional perturbations of specific proteins, supports in an unbiased, purely computational way the general mechanisms involving MTRNR2LXs.

### 3.7. Evaluating Hubs and Bottlenecks

In an interactome, hub nodes are easy to identify because they have numerous connections, whereas bottleneck nodes, which act as bridges between modules, are harder to detect. The main parameter used to identify bottlenecks is betweenness centrality, supported by closeness centrality, which reflects diffusion efficiency, and eigenvector centrality, which measures a node’s importance based on the number and quality of its connections. Bottlenecks lie on many shortest paths in the network and connect functional modules or clusters [96]. For this reason, proteins identified as bottlenecks are often essential for cell survival: removing them can fragment the network and disrupt vital biological processes [97]. Unlike hubs, which are important for network stability and functionality, bottlenecks regulate the flow of information [98]. A protein may be both a hub and a bottleneck, but many important bottlenecks have relatively few direct connections.

Although bottlenecks are often essential genes or proteins under strong selective pressure, their topological centrality remains a theoretical prediction. In dynamic systems such as cells, a protein’s bottleneck role may depend on factors like subcellular localization or half-life in a given phenotypic state, which static interactome maps may not capture [99,100]. Thus, topological parameters are powerful tools for generating functional hypotheses, but they are not absolute biological truths. Topology is a starting point for exploring possible interactions, not necessarily the order of events. Biological hierarchy is ultimately shaped by thermodynamics, especially binding affinity, which network topology alone cannot represent [101]. Therefore, a node with few connections but very high affinity may be more important than a highly connected hub with weak interactions.

Our identification of hub and bottleneck nodes relied on two pairs of topological parameters: degree and eigenvector centrality for hubs, and betweenness and closeness centrality for bottlenecks. Degree centrality reflects the number of a node’s connections, so nodes with high degrees are often considered hubs. Eigenvector centrality considers both the quantity and the significance of a node’s connections. Together, these metrics more reliably identify hub nodes that are essential for the stability and function of the protein–protein interaction network and may represent targets for therapeutic or functional studies.

Betweenness centrality measures how important a node is in connecting other nodes through the shortest paths. In protein–protein interaction networks, high betweenness suggests that a protein is important for information transfer and biological pathways. Closeness centrality measures how quickly a node can reach all other nodes in the network. Together, these complementary metrics identify bottleneck proteins, which act as control points or bridges in the interactome. Even without a high degree, their removal can fragment the network and disrupt communication between functional modules.

Using Cytoscape and the CentiScaPe plug-in, we calculated the four topological parameters for all 1033 nodes. Because of the large network size, we applied a common interactomics approach by selecting about 10% of the nodes. For each category, the top 100 nodes were selected and compared in pairs (degree vs. eigenvector; betweenness vs. closeness), and the common nodes in each pair were kept. Table 4 and Table 5 report the resulting hub and bottleneck nodes.

### 3.8. The Hub-and-Spoke Representation of Interactome-1033

**Figure 5:**
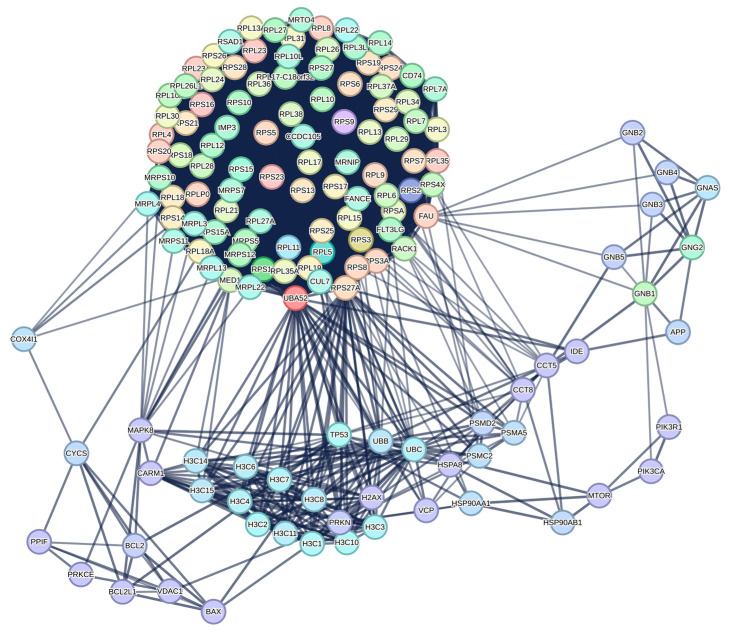
Hub and spoke representation of hub and bottleneck proteins from interactome-1033. The network was generated using STRING with confidence score ≥ 0.700 and exclusion of the text-mining channel. Topological parameters: 142 nodes, 4780 edges, average node degree 66.3, network diameter 3, network radius 2, characteristic path length 1.59, average local clustering coefficient 0.848, network density 0.486, network heterogeneity 0.550, network centralization 0.334, expected number of edges 1923, connected components 1, PPI enrichment *p*-value < 1.0 × 10^−16^. GNB1 and TP53 are highlighted as candidate high-centrality nodes within G-protein- and apoptosis-associated modules, respectively.

Previous analyses suggested that the interactome’s core nodes may follow a hub-and-spoke model, which would support many of our functional hypotheses. To validate a hub-and-spoke (“star”) architecture in a protein–protein interactome (PPI), one must identify a few central proteins (hubs) with many interactions and many peripheral proteins (spokes) that interact almost exclusively with their hub [91,92,93]. The nodes in Table 4 and Table 5 were submitted to STRING to generate the corresponding interactome, shown in Figure 5. This network contains 4708 protein–protein interactions, 88.36% of which are highly reliable, with confidence scores ranging from 0.700 to 0.999. Its architecture shows a classic hub-and-spoke pattern: hubs are connected to one another, whereas spokes are weakly connected to each other and mainly linked to the central hub module. In such architectures, the clustering coefficient is low for spokes and variable for hubs. If spokes interact only with the hub, they do not cluster together. Peripheral proteins in a hub-and-spoke network have neighborhood connectivity equal to the degree of the hub to which they are connected [102].

Spoke nodes should cluster tightly around individual hubs, forming distinct modules detectable by clustering algorithms. In a pure star, the average shortest path between any two spokes passes through the hub and is very low (~2), and the diameter is usually small (2 or 3). Hubs should also have high closeness values to enable rapid responses to cellular signals. Supplementary Dataset S4 lists these patterns and shows that the nodes are organized according to a hub-and-spoke model (see also the distribution patterns in the Supplements). These data indicate that the network is not random but organized around highly connected central nodes that coordinate communication among many peripheral nodes. This graph effectively summarizes the main functional activities in interactome-1033. It contains 924 enriched functional terms (Table S4), and the most significant Biological Processes correspond to standard cellular housekeeping functions (Figures S7 and S8, Table S5). It also shows enrichment for several mitochondrial functional terms (Table 6). Although these functions are more specific and less obvious, they remain meaningful and are expected to yield lower statistical values than broader biological functions. Table 7 lists all core proteins involved in the major mitochondrial molecular processes reported in Table 6. These nodes coincide with those already identified in interactome-1033 and discussed previously (Table 4 and Table 5). In the following paragraphs, we analyze their functional role within the topological context of the entire interactome.

### 3.9. Distribution of Topological Parameters

**Figure 6:**
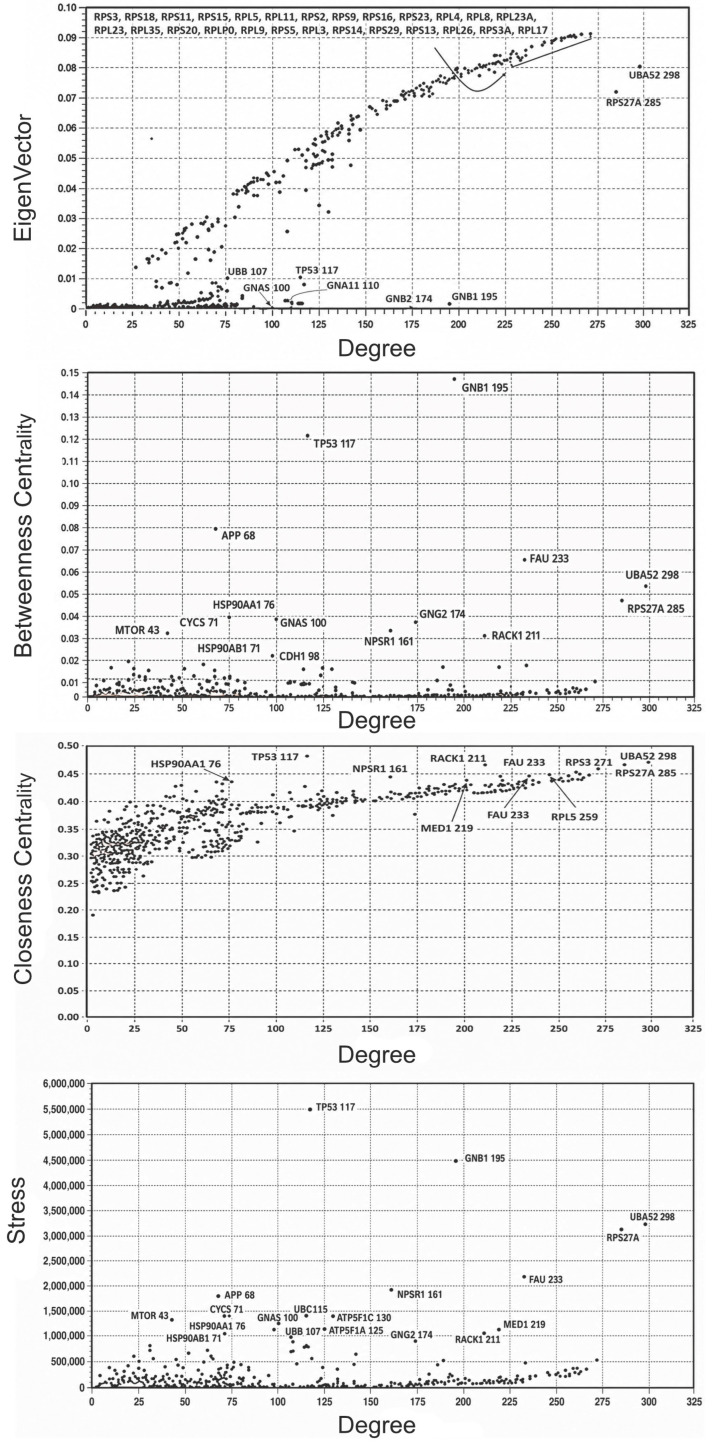
Topological distributions of interactome-1033. Distribution of four important topological parameters (Eigenvector centrality, Betweenness centrality, Closeness centrality, and Stress centrality) in the protein population forming the interactome-1033. The complete lists of these topological parameters are contained in the Supplementary Dataset S4.

Studying the distribution of topological parameters across the interactome helps clarify the cell’s functional and structural organization at the systems level. These analyses turn protein–protein interaction lists into biological maps, shifting the view from individual proteins to the network. Topology analysis can reveal a protein’s role from its network position, identify functional protein groups, and predict disease-related genes. The four centrality graphs in Figure 6 (Eigenvector, Betweenness, Closeness, and Stress) show the node distribution in interactome-1033 and highlight each node’s hierarchical importance.

In biomedical research, molecules and their functions are often reduced to hypothetical average entities, masking major differences among individual molecules in cells. Traditional analyses focus on differences between group means and controls, while overlooking variation within populations and individual responses. A more informative approach considers the full distributions of key topological traits and their interactions across affected populations, including biological subgroups defined by genotype or function. This distribution-aware view broadens the usable data, better reflects biological complexity, and treats heterogeneity as a resource for improving experimental design and for predicting and interpreting functional outcomes at both the group and individual levels. Analysis of the four distributions reveals several topological features:

1. Eigenvector Centrality (Node degree)—The graph shows a typical scale-free distribution: most proteins have few links, while a few hubs have very high degrees (over 200). The dominant nodes are UBA52 (298) and RPS27A (285), indicating that the “engine” of the network is focused on protein turnover and ribosome synthesis. Small mitochondrial proteins lie at the tail of the distribution (low degrees in the left corner), suggesting they are peripheral nodes that send specific signals to central hubs. Even low-degree nodes can be functionally important if they are specialized in critical roles. This is especially true for regulatory proteins that interact with key nodes controlling vital processes and strongly influence cell signaling.

2. Betweenness Centrality (intermediation)—This metric identifies bridges or bottlenecks that control information flow between network regions. Values are heavily skewed toward zero for most proteins, with a few exceptions such as TP53 (117) and HSP90AA1 (76). Because the network is highly modular, these proteins act as essential connectors between the small humanins module (MTRNR2LX) and stress-response and apoptosis pathways. Removing them would impair communication between MTRNR2LXs and the rest of the cell.

3. Closeness Centrality (interaction probability)—Closeness measures how quickly a protein can interact with others in the network. The distribution is right-shifted (0.4–0.5), typical of small-world networks. Despite the large number of proteins (1033), each node is only a few hops from the others. This suggests that signals from MTRNR2L8/12 can rapidly reach large cellular complexes, such as the ribosome or proteasome, through the identified mediators (FPR2 and G proteins).

4. Stress Centrality—This parameter is similar to Betweenness, indicating the traffic load passing through a node along the shortest paths. Peaks are observed for FAU (233) and RACK1 (211), showing that these proteins experience high communication stress. RACK1, a well-known scaffold coordinating signals from different pathways, appears to be a major terminal receiving input from G proteins activated by MTRNR2LXs. Overall, the topology describes a network in which MTRNR2LXs may act as peripheral sensors that, through the FPR2/G protein signaling interface, influence major hubs (ribosomes/ubiquitin) and global regulators (TP53/HSP90) to shape cell fate.

### 3.10. The Search for the Bridge

Given the architecture outlined, we must now functionally isolate the true “bridge” linking small mitochondrial proteins to large cellular complexes. Our data suggest three approaches: (A) perform a “Hierarchy of Control” (Bottleneck) analysis by comparing the Betweenness Centrality of FPR2 with that of G-proteins (GNB1, GNG2, GNAI2, GNB2); (B) if FPR2 has lower Betweenness Centrality than the G-proteins, this suggests the G-proteins integrate signals from various receptors, not only the MTRNR2LXs; (C) conversely, if FPR2 has high “Stress” or “Betweenness” centrality, this suggests the rest of the cell is the sole regulator of the mitochondrial module. A look at the table that ranks proteins by Betweenness Centrality (Supplementary Dataset S4) provides clear numerical evidence of the interactome hierarchy.

1—GNB1 is the key strategic bottleneck. It ranks first with the highest betweenness value (0.142). Although some proteins have more links (e.g., RPL5, ranked 259th), GNB1 lies on a high proportion of shortest paths between network regions. Thus, GNB1 is not just a partner but the bottleneck through which most communication between the MTRNR2LX module and the rest of the cell flows. Its high betweenness also identifies it as a vital hub and broker. In a “small-world” network, GNB1 enables MTRNR2L8/12 to reach distant hubs such as TP53 and APP in the fewest steps. Among the strongest broker nodes, TP53 (0.121) acts as the cell-cycle supervisor, supporting the idea that tiny humanin signals may be topologically connected to cell-survival-related modules. APP (0.079) ranks third, highlighting the interactome’s relevance to neuroprotection: APP is a crucial information hub and a bridge to pathology and stress.

2—Positioning of MTRNR2L8 and FPR2. Further down the list, MTRNR2L8 appears at 0.0086 (46/1033), far below GNB1, confirming its role as a peripheral signal initiator. FPR2 is even lower at 0.0025 (106/1033). MTRNR2L8, like MTRNR2L12, has low betweenness because it starts signaling; the signal then converges on GNB1, suggesting GNB1 may represent a high-centrality candidate interface. Pharmacological or genetic modulation of GNB1 should therefore produce broader cascading effects than direct intervention on small mitochondrial proteins. Links between GNB1 and nodes such as BCL2 (0.017, 18/1033) and BAX (0.006, 77/133) explain how the MTRNR2LX “survival” signal is routed to block cell death. Overall, the structure is “funnel-shaped”: numerous proteins, including MTRNR2LXs, feed into a few central hubs, GNB1, TP53, and APP, that regulate the response. MTRNR2L8 and 12, at the interactome periphery, send a signal detected by FPR2; from there it converges on GNB1, which distributes it to major regulators including TP53, APP, and the ubiquitin machinery (UBA52). GNB1 is therefore one of the network’s most influential proteins because it can affect distant processes.

Table 8 shows that GNB1 interacts with 189 proteins, confirming its role as a highly active signaling hub, consistent with its high betweenness centrality and broad partner network. These interactors are distributed across the network’s functional modules, where they participate in numerous signal-transduction and regulatory processes. Their wide distribution underscores how extensively GNB1 is broadly connected across multiple signaling modules.

Although topology does not always reflect biological reality, the model we are building is grounded in systems biology and generates high-probability hypotheses. If GNB1 interacts with 189 partners, its funnel-like position is the most statistically consistent explanation for the many pleiotropic effects reported in the literature.

### 3.11. The G-Protein Signaling Core

GNB1 interacts with nearly all other G-protein subunits, placing it at the core of signal transduction. It interacts with GNB2, GNB3, and GNB5, underscoring redundancy and complexity in dimer formation, and with GNG2, GNG3, GNG4, GNG5, GNG7, GNG8, GNG10, GNG11, GNG12, GNG13, GNGT1, and GNGT2 (GNG γ subunit). This diversity of gamma-subunit partners helps determine downstream signaling specificity and receptor localization. GNB1 also interfaces with several key receptors, including many G-protein-coupled receptors (GPCRs) relevant to this study:

- FPR2/FPR1: GNB1 directly interacts with both formylpeptide receptors, supporting the pathway linking MTRNR2LXs to the network.
- Neurotransmitter Receptors: GNB1 interacts with serotonin (HTR1A, HTR1B, HTR2C, etc.), dopamine (DRD1, DRD2, DRD3), and opioid (OPRM1, OPRD1) receptors, supporting its role in modulating neural responses, pain, and pleasure.
- Hormone and Metabolic Receptors: GNB1 binds somatostatin (SSTR2), melatonin (MTNR1A), and intestinal peptide receptors (GLP1R, GCGR, GIPR). Among downstream effectors, it directly interacts with PI3K, including PIK3CA, PIK3CB, PIK3CG, and PIK3R1. This pathway appears central to cell survival via AKT and likely explains the cytoprotective effect of MTRNR2LX.

In summary, GNB1 functions as the main sorting hub, receiving signals from multiple surface receptors, including FPR2 activated by MTRNR2LXs, and converting them into survival or metabolic responses. The analysis is highly precise because it is based on 189 proteins that represent all GNB1 interactions in our filtered interactome. This suggests we isolated a signaling system free of interference. However, the full list of GNB1-exclusive partners reveals additional insights.

1. A massive reception system (GPCR-centric)—Nearly all GNB1 partners are G-protein-coupled receptors (GPCRs). GNB1 does not generate “noise” through cytoskeletal or metabolic interactions; it is dedicated to translating extracellular signals. In this context, MTRNR2L8 and MTRNR2L12 [14,15] are not merely “mitochondrial proteins” but selective ligands that enter a system specialized for hormones and neurotransmitters.

2. The signaling “core”: PI3K and survival—Among the few non-receptor proteins on the list, PIK3CA, PIK3CB, PIK3CG, and PIK3R1 stand out as phosphoinositide 3-kinase (PI3K) subunits. Because these are among the few GNB1 effectors besides receptors, the main message of MTRNR2LXs, mediated by FPR2, is almost certainly cell survival through AKT.

3. Isolated strategic connections—Beyond receptors, GNB1 interacts with several key partners. APP [2], through its direct association with GNB1, supports the functional link between humanins and neuroprotection. FAU [103,104], a proapoptotic node and one of the few non-GPCR contact points, suggests a direct role in regulating cell death. KCNJ3/5/6/9 potassium channels (GIRK) are also involved, indicating that GNB1 influences cellular excitability. This helps explain GNB1’s high betweenness value (0.142) and its role as a central hub that collects inputs from numerous receptors, including those for MTRNR2Lx, and channels them to critical effectors such as PI3K, APP, and FAU. In a small-world network, GNB1 enables MTRNR2L8 and MTRNR2L12 to reach distant hubs such as TP53 or APP in a few steps. The information flow follows an hourglass architecture:

Numerous inputs → GNB1 → a few key outputs.

This helps explain how small mitochondrial peptides can produce broad systemic effects. GNB1 acts as a key mediator and modulator, supporting a coherent biological view in which function emerges from modules rather than solely from the peptide. The peptide is interchangeable: two paralogues, MTRNR2L8 and MTRNR2L12, with similar properties, can bind the same module and trigger identical responses.

The function lies within a module, such as apoptosis, while the peptide acts as a key that alters the module’s state. Their dependence is minimal, as they extend modules that could function independently in response to different stimuli. In silico knockout experiments, removing all MTRNR2LX, showed that module cohesion remained intact, indicating that the peptide is not core to the module. Its role is limited to network activation, not structural integrity. This peripheral localization suggests the cell has evolved subtle control systems—molecular “plugins” that modify output without affecting the nucleus or core system. Unlike structural mitochondrial proteins, MTRNR2L8 and MTRNR2L12 signaling converge at a single-entry point, the FPR2 receptor, where the FPR2-GNB1 axis acts as a survival signal. Rather than dispersing, the signal is funnelled through this receptor. FPR2 activation collapses the signal onto GNB1, a node with an intermediation value of 0.142, redirecting mitochondrial input to broader decision-making pathways. GNB1 turns a small peptide signal into a systemic response. Its binding to APP and TP53 underpins the neuroprotective and apoptosis-regulating roles of humanin-like proteins. The GNB1-APP link helps explain the long-reported neuroprotective effect, whose molecular bridge remained unclear. GNB1 acts as a shield, modulating MTRNR2LX signaling to counter APP toxicity. Its interactions with FAU and the PI3K pathway suggest it sustains survival signals, such as AKT, and suppresses apoptosis. Interactions with many GPCRs, including serotonin, dopamine, and opioid receptors, suggest these small proteins may alter cellular sensitivity to external stimuli through crosstalk. For example, a cell producing high levels of MTRNR2LX may respond differently to dopamine or somatostatin because GNB1 is already occupied or pre-activated by mitochondrial signals. Overall, the interactome reveals a communication network that controls cell fate under stress, forming a cytoprotective emergency circuit. When the cell senses a threat, MTRNR2L8/12 unblocks FPR2 and activates GNB1. As a strategic hub, GNB1 regulates critical pathways, including APP, PI3K, and TP53, guiding the cell toward resilience and repair rather than apoptosis.

#### MFN2 as an Indirect Stress Rheostat

An additional layer of mitochondrial regulation that may be relevant within this framework is represented by Mitofusin 2 (MFN2), a mitochondrial outer membrane GTPase involved in mitochondrial fusion, mitochondria–ER contact sites, mitochondria-associated membranes (MAMs), and mitophagy [105,106]. In our core–periphery model, MFN2 could serve as a structural and metabolic relay, functionally or indirectly linking the MTRNR2Lx FPR2/GNB1 peripheral axis to long-term adaptation through its network interactions with key mitochondrial quality-control nodes such as PRKN and VDAC1. In particular, activation of the PI3K/AKT downstream survival cascades via Humanin signaling could modulate mitochondrial dynamics and mitophagy through a topologically validated path involving the HSP90AA1 chaperone module, which directly interacts with PRKN within our network framework (combined score: 0.800). Since PRKN strongly interacts with both MFN2 (combined score: 0.991) and VDAC1 (combined score: 0.895), this HSP90AA1-mediated axis provides a robust physical and functional link through which peripheral survival signals can stabilize mitochondrial architecture [107,108]. Crucially, our network reveals a direct and prominent interaction between PRKN and the pro-apoptotic executioner BAX (combined score: 0.921). Within this network context, PRKN activation could function as a critical molecular switch that suppresses BAX-mediated mitochondrial outer membrane permeabilization, thereby buffering errant apoptotic engagement and prioritizing mitochondrial clearance or repair. This concerted functional interaction would favor MFN2-dependent mitochondrial fusion, reducing organelle fragmentation, stabilizing oxidative phosphorylation, and buffering the production of reactive oxygen species (ROS) under non-acute stress conditions [109]. By preserving the plasticity of the mitochondrial network and preventing a sudden, catastrophic release of cytochrome c, this mechanism ensures that the stress signal reaches the TP53 central hub in a controlled, attenuated manner. As a result, the cell is guided towards a survival phenotype associated with senescence and active reparative pathways, orchestrated by chromatin remodelers such as KMT2A/WDR5 and KAT6A, rather than towards irreversible apoptotic engagement. This model shifts the cytoprotective paradigm of nuclear humanins from a direct, stoichiometric mechanism of consumption to an indirect, system-level conservation of mitochondrial adaptability. In summary, mitofusin-2 constitutes a critical mechanistic nexus linking indirectly peripheral signaling, organelle architecture, and the nuclear transcriptional response under conditions of chronic stress [110,111,112].

### 3.12. Clustering Analysis of Interactome-1033

**Figure 7:**
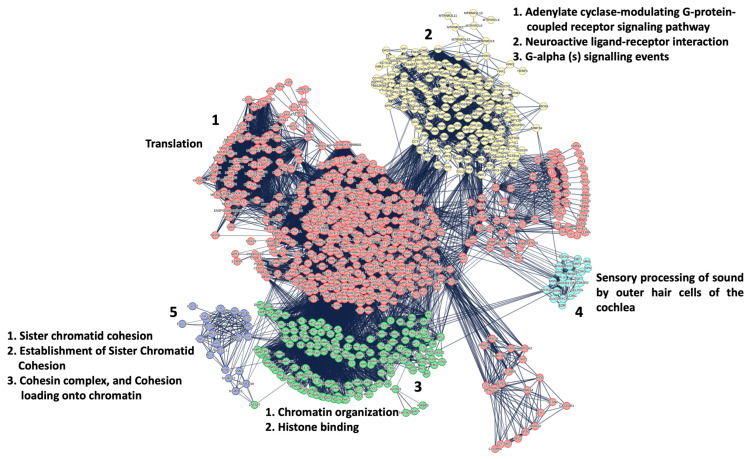
Cluster analysis of the interactome-1033. The K-means clustering analysis identified five functional modules, each represented by a distinct color. The main biological functions associated with each module are reported next to the corresponding cluster. The analysis was performed using STRING with K = 5.

Clustering analysis is a network science technique that divides a cell’s entire network of molecular interactions (interactome) into smaller, cohesive, and functionally similar groups called clusters or modules [113]. While the interactome shows how thousands of proteins interact, clustering identifies “communities” of proteins that work together to perform specific biological functions. Our goal is to extract functionally relevant modules from a highly complex system, such as the 1033-interactome. This should help us identify the functional regions where mitochondrial protein activity is focused and how they relate to one another. The analysis aims to identify groups of proteins that interact extensively among themselves but have few interactions with the rest of the network. If a protein’s function is unknown but it is part of a cluster where the other proteins are all involved in the same functional activity, we can use statistical methods to infer whether the unknown protein also performs that function (the concept of “function by association”). The entire population of interacting molecules is derived from a highly reliable database and rigorous network implementation procedures. This should produce high-quality clusters, with non-redundant results and low noise. Figure 7 shows the results of the clustering analysis.

The outcome is a “map” that visualizes the complex interactome as a set of distinct modules, each characterized by specific functions. We used a K value of 5 because it produced the most compact clusters and the most significant *p*-values, avoiding overly small clusters with very few nodes. The metabolic modules are all functionally coherent, and in Figure 7, we also show the connections between the clusters. It is visually clear that some key nodes maintain functional relationships among modules, while most constituent nodes interact closely. For example, the CCTx proteins in cluster 2 mediate numerous interactions with cluster 1, which uses the PIK3 and JAK groups of kinases to interact with clusters 2 and 4. This shows how information flows rapidly between clusters. Cellular life depends on normal metabolic processes, as represented by the metabolic relationships among the clusters. The interest in these subgraphs also comes from the fact that they contain the identified hub and bottleneck nodes, which, again in this analysis, are involved in the previously observed critical metabolic pathways.

Peculiar is the organization of the central module, which is also the largest. Understanding its specific organization and functions is essential, as various proteins that perform important functions within the cell operate within it. What we observe is a core–periphery organization. Core–periphery is a feature we sometimes observe in group-level relationships in biological networks, but it is not the only one [114]. It describes a scenario in which a compact group of central nodes captures an excessive number of network contacts. These peripheral node groups have fewer interconnections among themselves, although they are connected to the central core. In networking, mesoscale events describe subcellular processes spanning different length scales, from cells to large molecular complexes, in which groups of molecules self-organize to form large functional core structures [91,92,115,116]. This complex organization generates a richer, more diverse functional repertoire, whereas, as a single module, it would have performed simpler, more local operations. Below, we will analyze clusters that highlight activity related to mitochondria or MTRNR2LXs proteins.

**Figure 8:**
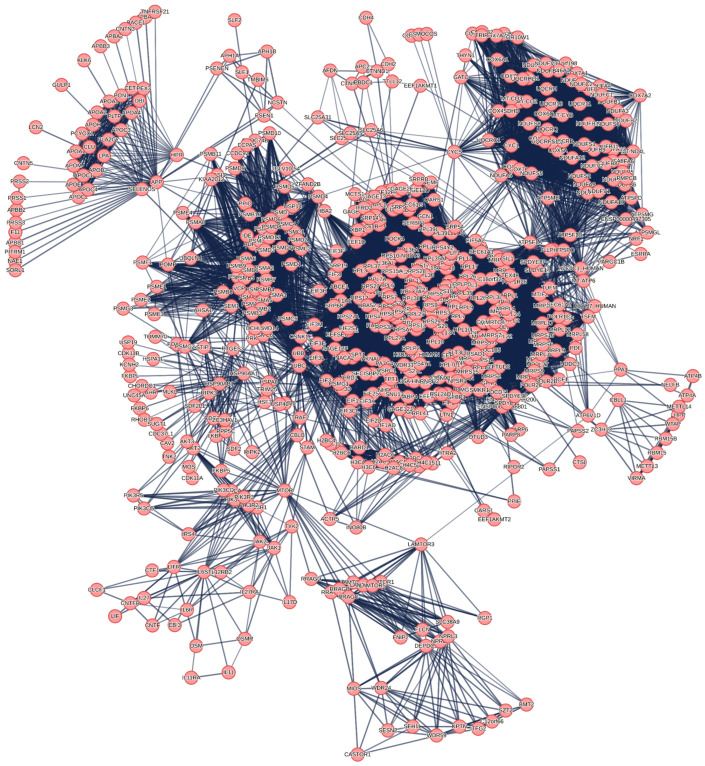
Cluster 1 represents the largest module identified by clustering analysis and is mainly enriched in essential cellular functions, including translation, mitochondrial activity, and core metabolic processes. Topological parameters: 618 nodes, 22,484 edges, average node degree 72.8, average local clustering coefficient 0.797, expected number of edges 7843, PPI enrichment *p*-value < 1.0 × 10^−16^.

Cluster 1, the largest—It primarily performs essential cellular activities (see Figure 8). STRING’s analysis of over 10,000 PubMed publications, conducted by in-house AI, shows that this cluster is associated with 1844 functional terms and co-occurs with hundreds of genes (Table S6, Figures S11 and S12 (GO Process) in the Supplements). Figure S11 shows that it regulates numerous mitochondrial activities necessary for normal mitochondrial function. There are no connections with MTRNR2LXs. This module includes most of the previously discussed hub proteins, notably several particularly important ones (UBA52, RPS27A, FAU, MED1, and RACK1), as well as all the hubs involved in the ribosomal system or regulating the electron transport chain and ATP production (see Table 9). It is also worth noting connections with certain central nervous system diseases, such as Parkinson’s, Alzheimer’s, Huntington’s, and Lou Gehrig’s disease, as highlighted by KEGG pathway enrichment (Figures S13 and S14 in the Supplements). However, a cluster of over 600 nodes is, in itself, a vast interactome with subgraphs that ensure stability and multifunctionality.

**Figure 9:**
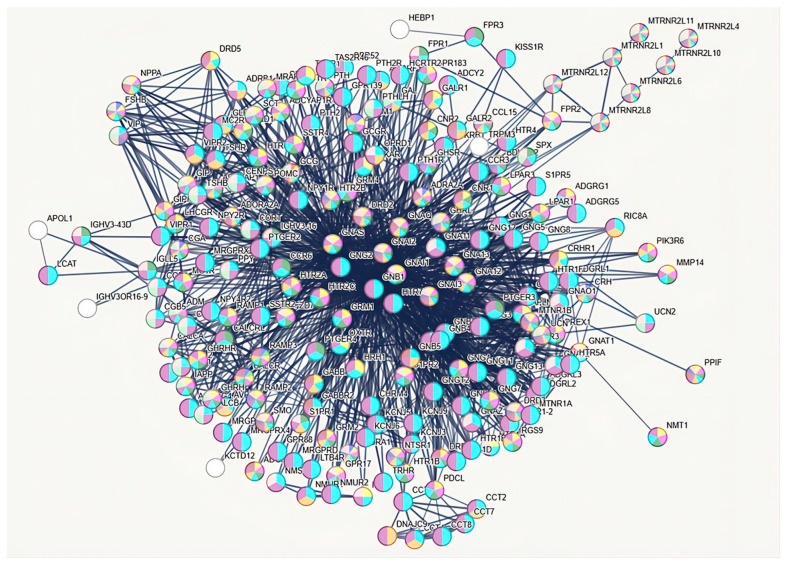
Interactome of Cluster no. 2. Topological parameters—number of nodes:213; number of edges: 1387; average node degree: 13; avg. local clustering coefficient: 0.697; expected number of edges: 41; PPI *p*-value < 1.0 × 10^−15^. Setting: the confidence score is 0.700 with no text mining. Some nodes are colored differently to distinguish their specific roles in the functional activities of MTRNR2LX proteins (see Table 10).

Cluster 2, the transmitter—This cluster shows activities related to G protein function, and again at a peripheral position, the group of MTRNR2LX proteins appears. Figure 9 shows the interactome of this cluster. It includes most of the previously mentioned bottleneck proteins, with the main ones being GNB1, GNG2, GNAI1, GNAS, IGHV3–16, GNAI2, GNAI3, GNB4, GNB2, GNB3, GNB5, and GNAQ, along with many other G proteins. The most-connected node is GNB1, with 189 interactors. G protein activity is central to the main functional processes in cluster 2. It displays 1073 enriched terms, mainly related to the biological functions of the large population of G proteins it contains. Figure S15 in the Supplements shows the most statistically significant functional activities. The cluster includes, at its periphery, the group of small MTRNR2LX proteins; it would therefore be interesting to understand how these small proteins interact with the members of this interactome and what effects they induce.

The “Show this node term in analysis table” function in STRING displays all the function terms associated with the selected node. Here, the protein examined is MTRNR2L8, a paralogue of MTRNR2L12, which is involved in 30 functional terms. Table 10 lists the most significant.

In GO analyses, MTRNR2L8’s involvement is marked by a specific color, shared by all nodes participating in the same process. Thus, node colors indicate shared functional activities driven by MTRNR2L8 and help identify the nodes involved and the flow linking them. MTRNR2LXs activity clearly extends across the cluster, involving nearly all nodes, although with different color patterns (see also Figure 9). In interactome-1033, only a few broad biological functions, such as adenylate cyclase modulation or G-protein signaling, reach Signal values near 10. Therefore, the Signal values observed for humanin-related processes, especially those between 1.0 and 2.0, show that these processes are not only statistically supported but also biologically meaningful. These values support both statistical confidence (FDR) and biological effect (strength is logarithmic), indicating a reliable process driven by MTRNR2L8.

**Figure 10:**
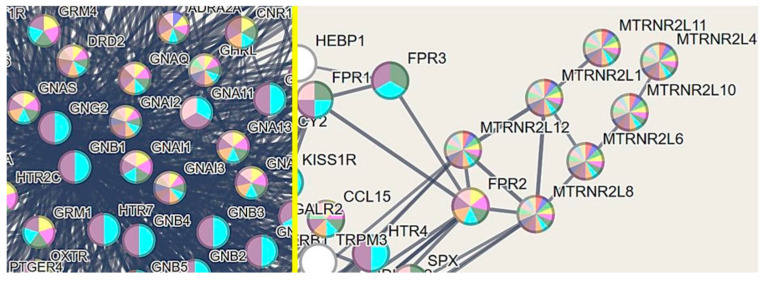
Functional relationships between MTRNR2Lx proteins and G-protein-associated nodes. Color coding indicates the functional categories shared by MTRNR2Lx proteins and their direct interactors, including FPR2, GNB1, GNG2, and GNAI2. Functional annotations correspond to the processes listed in Table 10.

Figure 10 shows a close-up view of the small humanins and their direct interactors, FPR2, GNB1, GNG2, and GNAI2, which are also key bottleneck nodes in both the full interactome and cluster 2. By comparing colors, the figure shows how different types of information from humanins are first distributed to these four interactors, in different ways, and then propagated across the cluster (see also Table 10). Because cluster nodes remain functionally linked to other external clusters, this information reaches nearly all functional modules of interactome-1033, spreading humanin-derived signals widely.

GNG2 and GNB1, beta-gamma signaling complexes, transmit information related to GO:0010469, regulation of signaling receptor activity (purple), GO:0050794, regulation of cellular process (light blue), both typical G-protein functions. While inhibitory processes pass through FPR2 (acting as a biochemical bridge) and GNAI2 (inhibitory subunit), including GO:1900118, negative regulation of the execution phase of apoptosis; GO:2000272, negative regulation of signaling receptor activity; GO:0048583, regulation of response to stimulus; GO:0044092, negative regulation of molecular function; GO:0043066, negative regulation of apoptotic process (only via GNAI2); and GO:0060548, negative regulation of cell death. Notably, FPR2 has lower betweenness centrality and stress values than the four G-proteins. This indicates that G-proteins also transmit signals from other receptors, not just from MTRNR2LXs. Humanins display a greater number of colors, but some associated processes are negligible and have not been accounted for. However, this data confirms what was previously stated.

**Figure 11:**
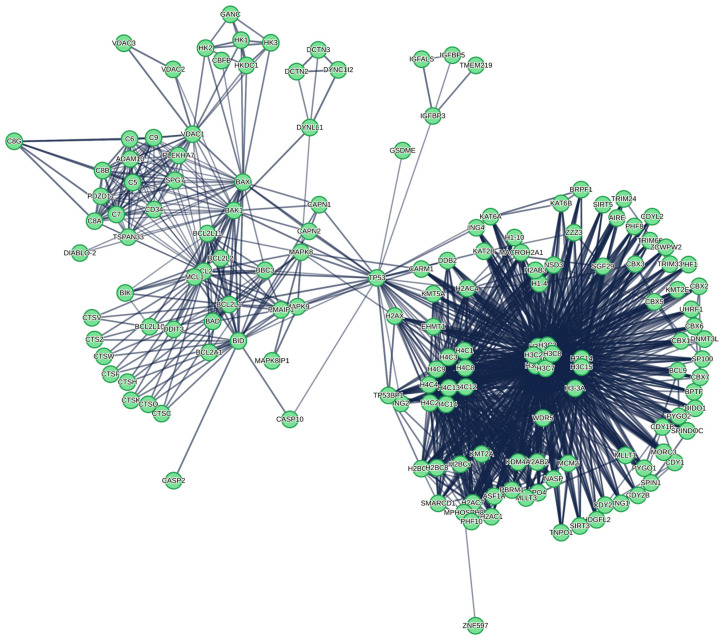
Interactome of cluster no. 3. Topological parameters: 157 nodes, 1497 edges, average node degree 19.1, average local clustering coefficient 0.845, expected number of edges 151, PPI enrichment *p*-value < 1.0 × 10^−16^. The network was generated using a confidence score ≥ 0.700 and excluding the text-mining channel.

Cluster 3, the world of TP53—Cluster 3 receives stress-derived signals from cluster 2. As shown in Figure 11, its interactome includes processes related to the nucleus, chromatin organization and remodeling, and mitochondrial membrane permeability.

**Figure 12:**
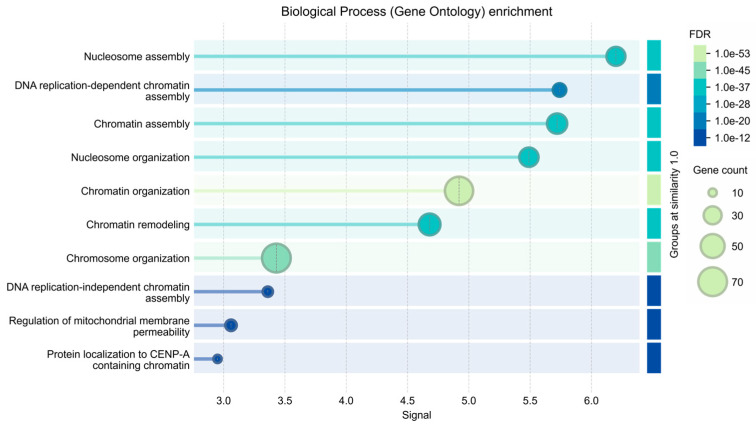
The 10 most significant functional relationships in cluster 3.

This profile suggests that TP53 is involved in multiple biological functions and acts as a pleiotropic hub. More broadly, this cluster reflects a hierarchy of cellular control that extends beyond transcription to influence both nuclear structural stability and mitochondrial bioenergetics. The extreme values of stress, betweenness, and bridging show that TP53 acts as both a guardian of the genome, initiating cell-cycle arrest or apoptosis, and as a physical coordinator between clusters and their functional compartments. Figure 12 shows the most important functional activities regulated by cluster 3. These activities are mainly responsible for regulating nuclear activities under the control of TP53.

Focusing on mitochondria, Table 11 summarizes the most significant mitochondria-related functional relationships among the 1074 enriched terms identified for this cluster.

These activities were enriched by an average of 25-fold (mean strength: 1.4) and were statistically significant. They involve the regulation of several mitochondrial functions in response to cellular stress, including cytochrome c release and control of mitochondrial membrane permeability. In this context, the high bridging and betweenness values of TP53 suggest that it manages the traffic between chromatin remodeling and mitochondrial permeability. TP53 does more than transmit chemical signals: its network position indicates that it coordinates the structural response. When chromatin is damaged, TP53 relays this information to mitochondria, helping regulate the mitochondrial membrane permeability threshold (MOMP). As a central regulator of chromatin organization, TP53 acts as an epigenetic sensor that translates the physical state of DNA into metabolic decisions.

Interestingly, the high eccentricity of MTRNR2Lx at the network periphery may allow these proteins to sense signals before they reach the core. They therefore act as cytoprotective antennae, detecting non-acute stresses, such as metabolic or mild oxidative stimuli, and relaying them to the center through G proteins. Their distance from the TP53 core may protect the system from abrupt activation, because the signal must pass through multiple steps before triggering apoptosis or extensive chromatin remodeling. This cluster has a bilobal, asymmetric architecture, with TP53 at the center between the two lobes, well connected, and with a clear bridging function. However, to understand its functions and how they occur, it is also necessary to evaluate the other nodes and their aggregations. TP53 has the highest centrality, but within this cluster, there are other highly eccentric nodes (see Supplementary Dataset S4). This suggests that these proteins could be exit points or niche regulators. These eccentric proteins could be those that perform highly specific, terminal tasks (e.g., a particular chromatin modification or the opening of a single membrane channel). TP53 decides, and these “cluster-peripheral” proteins execute the orders in an isolated region of the cell. Among the 157 nodes, we find:

1. “Remodelers” and Epigenetics (The heart of the cluster)—This is the most significant component. They are not just classical transcription factors but proteins that physically alter DNA accessibility:

- Histone Code Readers and Writers: a large group of histones (H1, H2A, H2B, H3, H4) and modifiers such as KAT2B (PCAF), KAT6A/B, KMT2A (MLL), and KDM4A.
- BRPF1/TRIM24/TRIM33 Complex: These are essential nodes. They act as “scaffolds” for complexes that acetylate TP53’s target genes.
- CBX proteins (1, 2, 3, 5, 6, 7): They are part of the Polycomb repressive complex (PRC1). Their presence indicates that the cluster not only “activates” but also maintains a delicate balance between silenced genes and genes ready to respond.

2. The Permeability Sensors (the bridge to the G-Proteins)—Here we find the molecular bridge connecting with the G-protein cluster and the MTRNR2Lx that we previously predicted:

- VDAC1, VDAC2, VDAC3: These are channels in the outer mitochondrial membrane. They regulate metabolite passage and serve as hubs where G-proteins and humanins exert their influence to prevent unnecessary cell death.
- HK1, HK2, HK3 (Hexokinase): These proteins physically bind to VDACs. Their presence in the TP53 cluster suggests that sugar metabolism is directly linked to nuclear survival decisions.

3. Apoptotic Effectors (“the armed wing”)—The entire BCL2 family (BAX, BAK1, BAD, BID, MCL1, BCL2L1) is included. It is important to note that TP53 is the only node that connects signals from chromatin remodelers (Section 1) with mitochondrial pore opening (Section 2) mediated by these proteins. This topologically organized architecture describes a highly regulated and resilient system:

- Noise Isolation: The significant distance (eccentricity) between humanins and the TP53 core suggests that the system is designed to ignore random fluctuations. Only persistent and coherent stress signals can travel from G-proteins to the nucleus.
- Vulnerability: If a pathogen or mutation affects the “bridges” connecting these two eccentric extremes (such as G-proteins or TP53 itself), communication between mitochondria and the nucleus would be disrupted, leading to an incoherent cellular response (e.g., the cell cannot undergo apoptosis when necessary, as seen in cancer, or it starts apoptosis prematurely, as in neurodegeneration). In conclusion, this is not just a functional cluster; it is a true command center that integrates three specific biological domains: epigenetics, apoptosis, and mitochondrial transport.

4. The “Gatekeepers” of Nuclear Transport—the cluster shows two proteins, IPO4 (Importin-4) and TNPO1 (Transportin-1), both with high eccentricity values and responsible for transporting transcription factors and histone modifiers into the nucleus. Their presence in the TP53 cluster suggests that communication between the G-protein module and the nucleus is not left to chance (e.g., passive diffusion) but is actively regulated. If humanins modulate G-protein activity, they could indirectly influence which importins “escort” TP53. A bridge between two distant clusters must pass through a transport system.

### 3.13. The Differing Functions of Mitochondrial Humanin (Mit-HN) and Nuclear Humanin-like

MTRNR2L8, 12, and FPR2/GNB1 are not the only actors involved. While MTRNR2L8 and 12 act peripherally by modulating external perturbations via FPR2/GNB1, mitochondria contain mitochondrial humanin, which is thought to act during acute stress. Under normal conditions, BCL2L1 continuously removes BAX from the mitochondrial membrane and returns it to the cytosol, where it remains inactive. This creates a dynamic inactivation cycle, although BAX still translocates to mitochondria at low levels. During acute stress, humanin in the mitochondria quickly binds to BAX, which stops the mitochondrial membrane from becoming permeable and prevents cytochrome c from being released. It also helps limit structural damage caused by free radicals, acting as an immediate buffering defense to prevent the sudden collapse of mitochondrial function. However, when stress exceeds mitochondrial synthetic capacity, which is limited by the small mitochondrial genome, higher-level regulation becomes necessary. If stress persists, humanin levels decline, and its defensive effect is exhausted. At that point, nuclear humanins (MTRNR2LX) become important. Because they are regulated by the nucleus, they can provide a broader and more sustainable response over time. From a small-world perspective, despite their peripheral position, nuclear humanins remain closely connected to control centers (TP53 and ribosomes), via strategic bridges, such as the FPR2/GNB1 complex. Their role is therefore regulatory rather than structural.

Thus, mit-HN serves as the body’s internal defense mechanism, managing acute stress, safeguarding mitochondrial integrity, and blocking immediate death signals (via BAX). The mitochondrion acts as the physiological detector of acute stress, and HN responds swiftly. The MTRNR2L8/12 →FPR2/GNB1 system establishes the peripheral defense system. It modulates external disruptions, communicates with functional modules, and coordinates long-term survival, all of which are mediated by PI3K/Akt signaling. This is an example of mitochondrion-to-nucleus retrograde signaling, in which a cytoplasmic effector of the retrograde signal “offloads” the stress response from the (exhausted) mitochondrion to the cell’s overall signaling system [117]. This system appears better suited to responding to chronic stress. It is also noteworthy that, while internal mit-humanin is quickly consumed, it receives metabolic support, for example, from sirtuins 3 and 5 (present in the interactome), which promote its restoration and enhance its function. Unfortunately, the view of the interactome remains static. It reflects the phenotypic state before the involvement of mit-HN, cytoplasmic MTRNR2LXs, and GNB1. In acute stress situations, we can only hypothesize that, since mit-HN is a consumable internal resource, if stress persists and supplies deplete, the system should intervene. Fortunately, some relevant information exists. Under cellular stress, substantial evidence indicates that mit-HN is also present outside mitochondria. The distinction between mitochondrial peptides and nuclear copies is a biochemical reality, documented in cell lines and by differential sequencing, confirming its accuracy. Mit-HN is produced within mitochondria but exerts much of its cytoprotective effect in the cytoplasm, where it interacts with pro-apoptotic proteins like Bax, Bid, and Bak, as well as IGFBP-3 (insulin-like growth factor), to modulate cell survival signals (functioning as a mitokine). So, it works via a dual mechanism:

1. Intracellularly: It shields the cell from oxidative stress and apoptosis.

2. Extracellularly (Receptor-mediated): It is secreted by the cell and acts on membrane receptors (such as the CNTFR/WSX-1/gp130 complex or the FPRL1 receptor) on other cells, activating cytoprotective signaling pathways (e.g., the JAK/STAT pathway). These support the hypothesis that HN can also serve as a signal to the nucleus and/or cytosol, prompting the expression and/or activation of nuclear MTRNR2LXs, thus enhancing its action. Therefore, a two-way communication system between mitochondria and the nucleus/cytosol can be envisioned, an idea that aligns well with the existing interactomic data.

## 4. Conclusions

The robust 1033-interactome model reveals that the peripheral placement of humanins (nuclear MTRNR2LXs) outlines their metabolic phenotype and regulatory roles in cellular energy balance and stress responses. Based on these topological analyses, we propose a comprehensive spatiotemporal model of humanin-mediated cellular resilience:

(1) **Perception**: Peripheral MTRNR2LXs act as “frontier sensors,” detecting non-acute metabolic and mild oxidative stress.
(2) **Mitochondrial Relay**: Downstream PI3K/AKT survival cascades stabilize Mitofusin-2 (MFN2), which acts as a dynamic “stress rheostat”. By promoting mitochondrial fusion and mitigating ROS production under chronic stress, MFN2 preserves organelle plasticity and prevents catastrophic cytochrome c release.
(3) **Transmission**: This controlled, MFN2-attenuated stress signal travels via G-proteins (GNB1, GNAI2) to the central TP53 core, setting its activation threshold and buffering against abrupt apoptotic over-activation.
(4) **Logistics & Restructuring**: Nuclear import proteins (IPO4, TNPO1) recruit histones and the chaperone ASF1A. In the nucleus, the KMT2A/WDR5 complex drives stable H3K4me3 chromatin methylation, regulated via non-degradative ubiquitination, while KAT6A mediates rapid, reversible acetylation.
(5) **Outcome**: Coordinated chromatin remodeling guides the cell toward a survival phenotype associated with protective senescence or SASP activation, avoiding irreversible apoptotic engagement.

Crucially, the transition from mitochondrial humanin (mit-HN) to nuclear MTRNR2LXs marks a strategic thermodynamic shift from a 1:1, rapidly depletable BAX-sequestration defense to an indirect, system-level conservation of mitochondrial adaptability. Operating via the FPR2/GNB1/MFN2 axis and catalytic PI3K/Akt signaling cascades, this network property sustains active resilience during chronic or neurodegenerative stress, establishing clear, testable molecular hypotheses for future in vitro and in vivo validation.

## Figures and Tables

**Table 1:** 42 experimentally validated LT interactors for humanins.

Experimentally Validated LT Interactor
APP, BAK1, BAX, BCL2L11, BH3, CDH2, CNTFR, CYCS, EEF1A1, FPR1, FPR2, FPR3, GNG2, HSP90AA1, IGFBP3, IL27RA, IL6ST, MPHOSPH8, MTRNR2L1, MTRNR2L10, MTRNR2L11, MTRNR2L12, MTRNR2L3, MTRNR2L4, MTRNR2L6, MTRNR2L7, MTRNR2L8, NPRL3, PPA1, PRDX1, PSMA5, PXDNL, RBM38, SLF1, SMC3, TRIM11, TRIM2, TRIM25, TSFM, USP40, VIRMA, VSTM2L

**Table 2:** Comparison between “experimentally determined PPIs” of Interactome 1041 and 1033.

Confidence Score	Experimental Channel of Interactome-1041 (Number of Validations)	%	Experimental Channel of Interactome-1033 (Number of Validations)	%
0.900	891	0.33	9717	35.91
0.800	615	0.23	5930	21.91
0.700	489	0.18	3086	11.40
TOTAL	1995	0.74	18,733	69.22

Note: Interactome-1041 has a total of 267,231 interactions. Interactome-1033 contains 27,059 interactions (90% less). The details are in the Supplementary Dataset S4.

**Table 3:** Summary of interactome-1033 topological parameter statistics.

Number of Nodes	1033
Number of edges	27,059
Average number of neighbors (average node degree)	52.4
Network diameter	7
Network radius	4
Characteristic path length	2.991
Average local clustering coefficient	0.761
Network density	0.056
Network heterogeneity	1.127
Network centralization	0.233
Connected components	1

Note: Calculations performed by Cytoscape.

**Table 4:** HUB-NODES of Interactome-1033.

Hub-Nodes
CCDC105, CD74, CUL7, FANCE, FAU, FLT3LG, GNB1, IMP3, MED1, MRNIP, MRPL13, MRPL22, MRPL3, MRPL4, MRPS10, MRPS11, MRPS12, MRPS5, MRPS7, MRTO4, RACK1, RPL1, RP*L10*A, RP*L10*L, RPL11, RPL12, RPL13, RPL13A, RPL14, RPL15, RPL17, RPL17-C18orf32, RPL18, RPL18A, RPL19, RPL21, RPL22, RPL23, RPL23A, RPL24, RPL26, RPL26L1, RPL27, RPL27A, RPL28, RPL29, RPL3, RPL30, RPL31, RPL34, RPL35, RPL35A, RPL36, RPL37A, RPL38, RPL3L, RPL4, RPL5, RPL6, RPL7, RPL7A, RPL8, RPL9, RPLP0, RPS10, RPS11, RPS13, RPS14, RPS15, RPS15A, RPS16, RPS17, RPS18, RPS19, RPS2, RPS20, RPS21, RPS23, RPS24, RPS25, RPS26, RPS27, RPS27A, RPS28, RPS29, RPS3, RPS3A, RPS4X, RPS5, RPS6, RPS7, RPS8, RPS9, RPSA, RSAD1, UBA52

**Table 5:** Bottleneck nodes of interactome-1033.

Bottleneck Nodes
APP, BAX, BCL2, BCL2L1, CARM1, CCT5, CCT8, COX4I1, CYCS, GNAS, GNB1, GNB2, GNB3, GNB4, GNB5, GNG2, H2AX, H3C1, H3C10, H3C11, H3C14, H3C15, H3C2, H3C3, H3C4, H3C6, H3C7, H3C8, HSP90AA1, HSP90AB1, HSPA8, IDE, MAPK8, MTOR, PIK3CA, PIK3R1, PPIF, PRKCE, PRKN, PSMA5, PSMC2, PSMD2, TP53, UBB, UBC, VCP, VDAC1

**Table 6:** Selective enrichment of mitochondrial functions using the hub-and-spoke model.

Term	Description	Count in Network	Strength	Signal	FDR
Biological Process (GO)					
GO:0032543	Mitochondrial translation	9 of 112	1.05	1.01	4.97 × 10^−5^
GO:0051881	Regulation of mitochondrial membrane potential	7 of 71	1.14	0.89	0.00028
GO:0046902	Regulation of mitochondrial membrane permeability	6 of 63	1.12	0.71	0.0017
GO:0001836	Release of cytochrome c from mitochondria	4 of 21	1.42	0.65	0.0042
GO:0090199	Regulation of release of cytochrome c from mitochondria	5 of 47	1.17	0.61	0.0050
GO:0010821	Regulation of mitochondrion organization	8 of 153	0.86	0.60	0.0033
GO:0006839	Mitochondrial transport	8 of 153	0.86	0.60	0.0033
GO:0008637	Apoptotic mitochondrial changes	5 of 58	1.08	0.52	0.0107
Cellular Component (GO)					
GO:0005761	Mitochondrial ribosome	9 of 90	1.14	1.35	1.51 × 10^−6^
GO:0098798	Mitochondrial protein-containing complex	13 of 296	0.78	0.97	1.25 × 10^−5^
GO:0005741	Mitochondrial outer membrane	10 of 209	0.82	0.83	0.00014
Reactome Pathways					
HSA-5368286	Mitochondrial translation initiation	9 of 88	1.15	1.40	8.91 × 10^−7^
HSA-111457	Release of apoptotic factors from the mitochondria	2 of 6	1.67	0.60	0.0071
Subcellular Localization (Compartments)					
GOCC:0005757	Mitochondrial permeability transition pore complex	4 of 11	1.70	1.05	0.00015
GOCC:0005741	Mitochondrial outer membrane	9 of 152	0.91	0.89	0.00011
GOCC:0031315	Extrinsic component of mitochondrial outer membrane	2 of 2	2.14	0.58	0.0089

Note: Graphs relative to other features of selected enrichments and related statistics, are available in the Supplements as Figures S9 and S10.

**Table 7:** Central proteins involved in the molecular processes listed in Table 6.

HUB Node	Bottleneck Node
MRPS10, MRPL4, MRPS7, MRPL3, MRPS11, MRPS5, MRPS12, MRPL13, MRPL22, UBA52, RPS27A, RACK1.	APP, BAX, BCL2, BCL2L1, COX4I1, CYCS, HSP90AA1, MAPK8, MTOR, PPIF, PRKN, TP53, UBB, UBC, VCP, VDAC1.

**Table 8:** Interactors of GNB1.

Interactors of GNB1
ADCYAP1, ADCYAP1R1, ADGRD1, ADGRF1, ADGRG1, ADGRG3, ADGRG4, ADGRG5, ADGRL1, ADGRL2, ADGRL3, ADM, ADM2, ADORA1, ADORA2A, ADRA2A, APLNR, APP, AVPR2, BDKRB1, BDKRB2, CALCA, CALCB, CALCR, CALCRL, CCKAR, CCL15, CCR3, CCR5, CCR6, CCT5, CENPS-CORT, CGA, CGB3, CGB5, CGB8, CHRM4, CNR1, CNR2, CORT, CRH, CRHR1, CRHR1–2, DRD1, DRD2, DRD3, DRD4, FAU, FPR1, FPR2, FZD7, GABBR1, GABBR2, GAL, GALR1, GALR2, GCG, GCGR, GHRH, GHRHR, GHRL, GHSR, GIP, GIPR, GLP1R, GLP2R, GNA11, GNA12, GNA13, GNAI1, GNAI2, GNAI3, GNAO1, GNAQ, GNAS, GNAT1, GNAT2, GNAZ, GNB2, GNB3, GNB5, GNG10, GNG11, GNG12, GNG13, GNG2, GNG3, GNG4, GNG5, GNG7, GNG8, GNGT1, GNGT2, GPR139, GPR17, GPR183, GPR52, GPR88, GRM2, GRM4, HCRTR2, HRH1, HTR1A, HTR1B, HTR1D, HTR1E, HTR1F, HTR2A, HTR2B, HTR2C, HTR4, HTR5A, HTR6, HTR7, IAPP, IGHV3–16, IGLL5, KCNJ3, KCNJ5, KCNJ6, KCNJ9, CTD12, LHCGR, LPAR1, LPAR3, LTB4R, MC1R, MC2R, MRAP, MRGPRD, MRGPRX1, MRGPRX2, MRGPRX4, *MTRNR1*A, *MTRNR1*B, MTRNR2L12, MTRNR2L8, NMS, NMU, NMUR1, NMUR2, NPSR1, NPY1R, NPY2R, NPY4R2, NTSR1, OPRD1, OPRM1, OXTR, PDCL, PIK3CA, PIK3CB, PIK3CG, PIK3R1, PIK3R5, POMC, PPY, PREX1, PTGER2, PTGER3, PTGER4, PTH, PTH1R, PTH2, PTH2R, PTHLH, RAMP1, RAMP2, RAMP3, RGS9, S1PR1, S1PR2, S1PR3, S1PR5, SCT, SCTR, SMO, SPX, SSTR2, SSTR4, TACR1, TAS2R46, TRHR, TRPM3, TSHB, TSHR, UCN, VIPR1, VIPR2

**Table 9:** Activities related to mitochondria of cluster no. 1.

Term	Description	Count in Network	Strength	Signal	FDR
Biological Process (GO)					
GO:0042775	Mitochondrial ATP synthesis coupled electron transport	62 of 92	1.33	6.94	1.72 × 10^−50^
GO:0042776	Proton motive force-driven mitochondrial ATP synthesis	43 of 64	1.33	5.57	1.70 × 10^−34^
GO:0006120	Mitochondrial electron transport, NADH to ubiquinone	31 of 46	1.33	4.45	1.40 × 10^−24^
GO:0032981	Mitochondrial respiratory chain complex I assembly	28 of 61	1.17	3.26	8.48 × 10^−19^
GO:0006123	Mitochondrial electron transport, cytochrome c to oxygen	18 of 23	1.40	3.10	8.25 × 10^−15^
GO:0032543	Mitochondrial translation	36 of 112	1.01	2.97	3.60 × 10^−20^
GO:0140053	Mitochondrial gene expression	38 of 143	0.93	2.63	5.80 × 10^−19^
GO:0033108	Mitochondrial respiratory chain complex assembly	30 of 98	0.99	2.55	3.30 × 10^−16^
GO:0006122	Mitochondrial electron transport, ubiquinol to cytochrome c	12 of 13	1.47	2.28	3.03 × 10^−10^
GO:0007005	Mitochondrion organization	48 of 445	0.54	1.23	1.86 × 10^−10^
Cellular Component (GO)					
GO:0005746	Mitochondrial respirasome	63 of 94	1.33	7.01	8.80 × 10^−52^
GO:0098798	Mitochondrial protein-containing complex	109 of 296	1.07	5.52	5.41 × 10^−70^
GO:0005743	Mitochondrial inner membrane	114 of 502	0.86	3.63	9.37 × 10^−55^
Reactome Pathways					
HSA-5368287	Mitochondrial translation	36 of 94	1.09	3.52	1.36 × 10^−23^
HSA-5389840	Mitochondrial translation elongation	34 of 88	1.09	3.42	2.12 × 10^−22^
Wiki Pathways					
WP111	Electron transport chain: OXPHOS system in mitochondria	68 of 103	1.32	7.20	2.08 × 10^−55^

**Table 10:** Activities related to humanins in cluster no. 2.

Term	Description	Count in Network	Strength	Signal	FDR
Biological Process (GO)					
GO:1900118	Negative regulation of execution phase of apoptosis	8 of 20	1.57	1.90	3.65 × 10^−8^
GO:0010469	Regulation of signaling receptor activity	15 of 179	0.89	1.30	2.46 × 10^−7^
GO:2000272	Negative regulation of signaling receptor activity	9 of 67	1.09	1.18	8.27 × 10^−6^
GO:0010646	Regulation of cell communication	97 of 3355	0.43	1.15	3.13 × 10^−19^
GO:0009966	Regulation of signal transduction	83 of 2978	0.41	1.05	9.31 × 10^−15^
GO:0048583	Regulation of response to stimulus	97 of 3931	0.36	0.96	1.25 × 10^−14^
GO:0050794	Regulation of cellular process	207 of 11,025	0.24	0.89	2.33 × 10^−40^
GO:0065009	Regulation of molecular function	75 of 3085	0.35	0.85	4.68 × 10^−10^
GO:0065007	Biological regulation	208 of 12,385	0.19	0.79	5.81 × 10^−32^
GO:0044092	Negative regulation of molecular function	34 of 1143	0.44	0.74	8.07 × 10^−6^
GO:0043066	Negative regulation of apoptotic process	25 of 891	0.41	0.55	0.00069
GO:0060548	Negative regulation of cell death	27 of 1016	0.39	0.53	0.00078
Molecular Function (GO)					
GO:0030545	Signaling receptor regulator activity	39 of 552	0.82	2.05	1.62 × 10^−17^
GO:0005102	Signaling receptor binding	74 of 1499	0.66	1.88	2.12 × 10^−26^
GO:0048019	Receptor antagonist activity	7 of 30	1.33	1.23	1.37 × 10^−5^
Local Network Cluster (STRING)					
CL:38222	Humanin family	7 of 8	1.91	2.10	1.42 × 10^−8^

Note: The table lists the processes in which humanins are most significantly involved. GO-terms are color-coded. This way, we can visualize how many processes each node of the interactome (Figure 9) is involved in, together with MTRNR2L8.

**Table 11:** Activities related to mitochondria of cluster no. 3.

Term	Description	Count in Network	Strength	Signal	FDR
Biological Process (GO)					
GO:0046902	Regulation of mitochondrial membrane permeability	15 of 63	1.48	3.06	4.17 × 10^−14^
GO:0008637	Apoptotic mitochondrial changes	14 of 58	1.48	2.90	3.44 × 10^−13^
GO:0001836	Release of cytochrome c from mitochondria	10 of 21	1.78	2.79	1.66 × 10^−11^
GO:0090199	Regulation of release of cytochrome c from mitochondria	11 of 47	1.47	2.29	3.35 × 10^−10^
GO:0090200	Positive regulation of release of cytochrome c from mitochondria	9 of 27	1.62	2.21	2.27 × 10^−9^
GO:0035794	Positive regulation of mitochondrial membrane permeability	9 of 32	1.55	2.06	7.32 × 10^−9^
GO:0010822	Positive regulation of mitochondrion organization	11 of 76	1.26	1.77	2.17 × 10^−8^
GO:1902686	Mitochondrial outer membrane permeabilization involved in programmed cell death	6 of 17	1.65	1.47	2.98 × 10^−6^
GO:0010821	Regulation of mitochondrion organization	13 of 153	1.03	1.44	1.59 × 10^−7^
GO:1902108	Regulation of mitochondrial membrane permeability involved in apoptotic process	6 of 22	1.53	1.32	9.47 × 10^−6^
GO:1902110	Positive regulation of mitochondrial membrane permeability involved in apoptotic process	5 of 15	1.62	1.16	4.74 × 10^−5^
GO:0007005	Mitochondrion organization	19 of 445	0.73	1.06	9.46 × 10^−7^
GO:0097345	Mitochondrial outer membrane permeabilization	4 of 12	1.62	0.89	0.00057
GO:0051881	Regulation of mitochondrial membrane potential	7 of 71	1.09	0.87	0.00028
Cellular Component (GO)					
GO:0005741	Mitochondrial outer membrane	18 of 209	1.03	1.98	2.01 × 10^−11^
GO:0005740	Mitochondrial envelope	25 of 802	0.59	0.96	4.14 × 10^−7^

## Data Availability

The original contributions presented in this study are included in the article/Supplementary Material. Further inquiries can be directed to the corresponding author(s).

## Supplementary Materials

The following supporting information can be downloaded at: https://www.mdpi.com/article/10.3390/biom16070981/s1. Supplementary Dataset S1. Experimentally validated low-throughput (LT) interactors for Humanin/Humanin-like (MTRNR2Lx) proteins and associated modules; Supplementary Dataset S2. Node degree statistics for the 1041-node interactome (confidence score 0.15, all channels open) and the 1033-node interactome (confidence score 0.70, text-mining disabled); Supplementary Dataset S3. Edge-level interaction distribution across STRING evidence channels for the 1041-node interactome (confidence score 0.15, all seven channels open) and the 1033-node interactome (confidence score 0.70, text-mining disabled); Supplementary Dataset S4. Node-level topological and centrality metrics for the 1033-node interactome (confidence score 0.70, text-mining disabled). Figure S1. Interactome-1041; Figure S2. The most significant biological GO Processes of the interactome in Figure S1; Figure S3. The most significant KEGG Processes of the interactome in Figure S1; Figure S4. Interactome-1041with a coefficient score of 0.700; Figure S5. The most significant biological GO Processes of the interactome-1041; Figure S6. The most significant KEGG Processes of interactome-1041; Figure S7. Gene counts for the most significant Biological Process (Gene Ontology) terms enriched in the hub-and-spoke model. Bubble size indicates gene count, and color indicates FDR; Figure S8. Signal values for the most significant Biological Process (Gene Ontology) terms enriched in the hub-and-spoke model. Bubble size indicates gene count, and color indicates FDR; Figure S9. Signal values of selected mitochondria-related enriched terms in the hub-and-spoke model; Figure S10. Gene counts of selected mitochondria-related enriched terms in the hub-and-spoke model; Figure S11. Signal values of biological processes of cluster 1; Figure S12. Count of genes of biological processes in cluster 1; Figure S13. Signal values in KEGG pathways of cluster 1; Figure S14. Gene counts in KEGG pathways of cluster 1; Figure S15. Significant functional activities of Cluster 2. Table S1. Enrichment of functional terms of Interactome_1041 with conf. score 0.15 and all channels open; Table S2. Mitochondrial gain control system; Table S3. Enrichment of functional terms of Interactome_1041 with conf.score 0.700 and no Text-mining; Table S4. Enrichment of functional terms of hub-and-spoke with conf. score 0.700; Table S5. Main biological process driven by hub-and-spoke model; Table S6. Enrichment of functional terms of cluster 1 (conf.score 0.700).

## Abbreviations

AKT	Protein Kinase B
APP	Amyloid Precursor Protein
BAX	BCL2 Associated X Protein
BioGRID	Biological General Repository for Interaction Datasets
CYCS	Cytochrome c, Somatic
FPR2	Formyl Peptide Receptor 2
GNAI2	G Protein Subunit Alpha i2
GNB1	G Protein Subunit Beta 1
GNG2	G Protein Subunit Gamma 2
GO	Gene Ontology
GPCR	G Protein-Coupled Receptor
HN	Humanin
IDP	Intrinsically Disordered Protein
KEGG	Kyoto Encyclopedia of Genes and Genomes
LT	Low-Throughput
MINT	Molecular INTeraction database
MTRNR2	Mitochondrially encoded 16S rRNA
MTRNR2L	MTRNR2-like genes (Humanin-like peptides)
PI3K	Phosphoinositide 3-Kinase
PPI	Protein–Protein Interaction
PPIN	Protein–Protein Interaction Network
R^2^	Coefficient of Determination
STRING	Search Tool for the Retrieval of Interacting Genes/Proteins
TP53	Tumor Protein p53
