# Supplementary material for: Network Topology and Interactomic Analysis Reveal the Regulatory Framework of the Humanin Protein Family (MTRNR2Lx Class)
Source: Biomolecules. 2026 Jul 3;16(7):981. doi: 10.3390/biom16070981 (PMC13406609; doi:10.3390/biom16070981)
Supplement: Supplementary file 1 [file biomolecules-16-00981-s001.zip › SUPPLEMENTARY FIGURES AND TABLES.pdf]

SUPPLEMENTS TO

Network topology and interactomic analysis reveal the regulatory framework of the Humanin protein family (MTRNR2Lx class).

Mohd Shahzaib [1], Domenico Aprile [2*], Gianluigi Laporta [1], Umberto Galderisi [1,3] and Giovanni Colonna [4*]

[1] Department of Experimental Medicine, Biotechnology and Molecular Biology Section, Luigi Vanvitelli Campania University, 80138, Naples, Italy
[2] Department of Life Sciences, Health and Health Professions, Link Campus University, 00165, Rome, Italy
[3] Genome and Stem Cell Center (GENKÖK), Erciyes University, Kayseri, Turkey
[4] Medical Informatics Unit - AOU L. Vanvitelli, Università della Campania, 80138 Naples, Italy.

* Correspondence:
Domenico Aprile: d.aprile@unilink.it
Giovanni Colonna: giovanni.colonna@unicampania.it

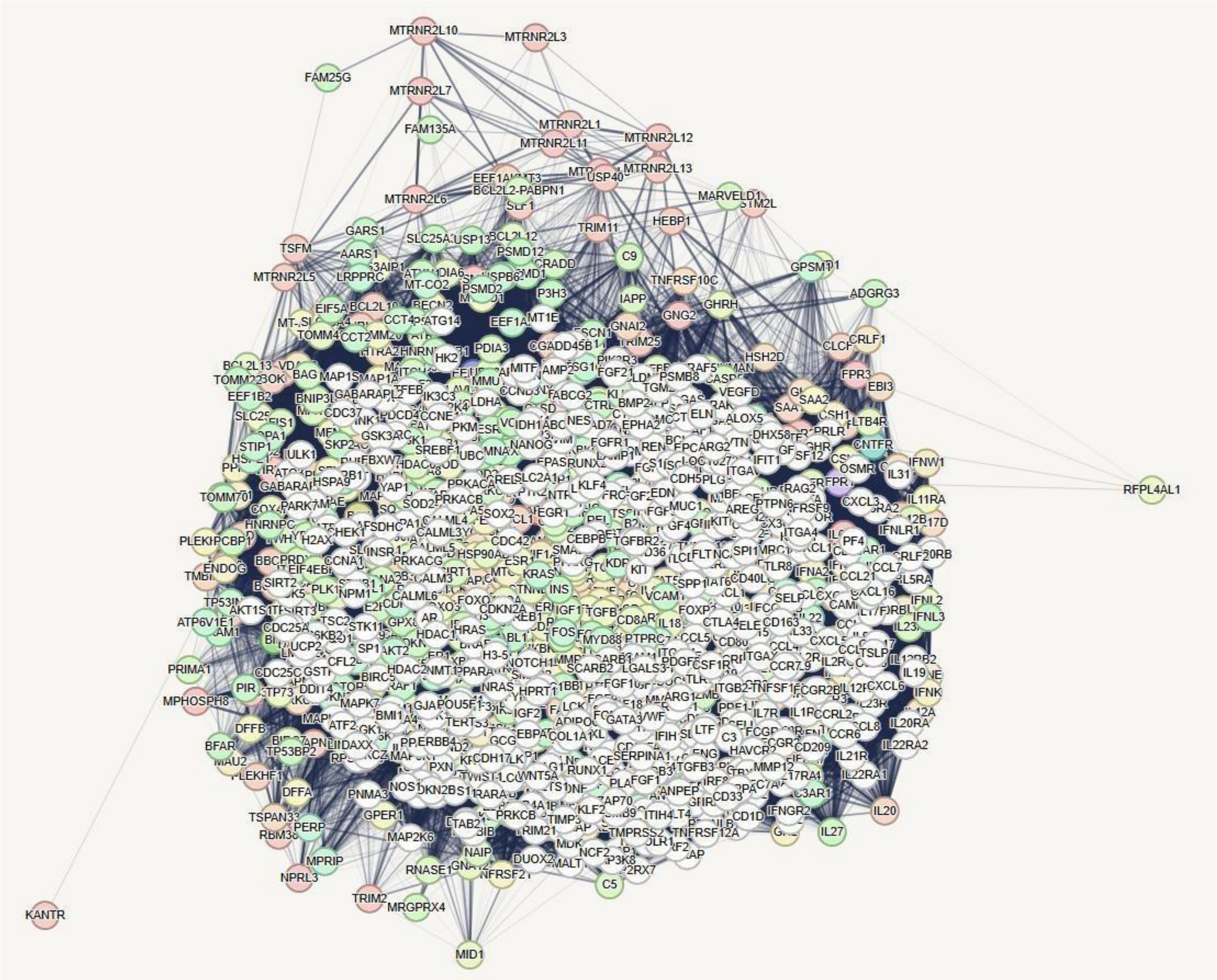

Figure S1 – Interactome-1041- The figure shows the enriched interactome derived from **Table 1** related to humanin interactors. The main topological parameters are confidence score 0.150; number of nodes: 1041; number of edges: 267,931; average node degree: 514; average local clustering coefficient: 0.731; expected number of edges: 119,469; PPI enrichment p-value: < 1.0e-16. All seven channels are open. Enrichment: 500 first-order and 500 second-order proteins. The second-order proteins serve as mediators, facilitating interactions between nodes.

This setting aims to capture all the molecular knowledge related to these proteins, as reported in 10,000 reference publications selected by STRING AI. The varying edge thicknesses emphasize the reliability of the interaction. The high average node degree suggests a very high number of false interactions (avg. node degree, 514). **Supplementary Dataset S3** shows the overall distribution and, in particular, that of the "Experimental" channel. Humanins are located peripherally, at the upper edge of the interactome.

Table S1 - Enrichment of functional terms of Interactome_1041 with conf. score 0.15 and all channels open

Biological Process (Gene Ontology)	download	*3461 GO-terms significantly enriched; file-format: tab-delimited*
Molecular Function (Gene Ontology)	download	*300 GO-terms significantly enriched; file-format: tab-delimited*
Cellular Component (Gene Ontology)	download	*248 GO-terms significantly enriched; file-format: tab-delimited*
Reference Publications (PubMed)	download	*10000 publications significantly enriched; file-format: tab-delimited*
Local Network Cluster (STRING)	download	*247 clusters significantly enriched; file-format: tab-delimited*
KEGG Pathways	download	*210 pathways significantly enriched; file-format: tab-delimited*
Reactome Pathways	download	*965 pathways significantly enriched; file-format: tab-delimited*
WikiPathways	download	*484 pathways significantly enriched; file-format: tab-delimited*
Disease-gene Associations (DISEASES)	download	*527 diseases significantly enriched; file-format: tab-delimited*
Tissue Expression (TISSUES)	download	*734 tissues significantly enriched; file-format: tab-delimited*
Subcellular Localization (COMPARTMENTS)	download	*331 compartments significantly enriched; file-format: tab-delimited*
Human Phenotype (Monarch)	download	*1576 phenotypes significantly enriched; file-format: tab-delimited*
Annotated Keywords (UniProt)	download	*110 keywords significantly enriched; file-format: tab-delimited*
Protein Domains (Pfam)	download	*11 domains significantly enriched; file-format: tab-delimited*
Protein Domains and Features (InterPro)	download	*162 domains significantly enriched; file-format: tab-delimited*
Protein Domains (SMART)	download	*47 domains significantly enriched; file-format: tab-delimited*
**All enriched terms** (without PubMed)	download	*9413 enriched terms in 15 categories; file-format: tab-delimited*
Selected terms only	download	*no enriched terms selected (click on any term above to select)*

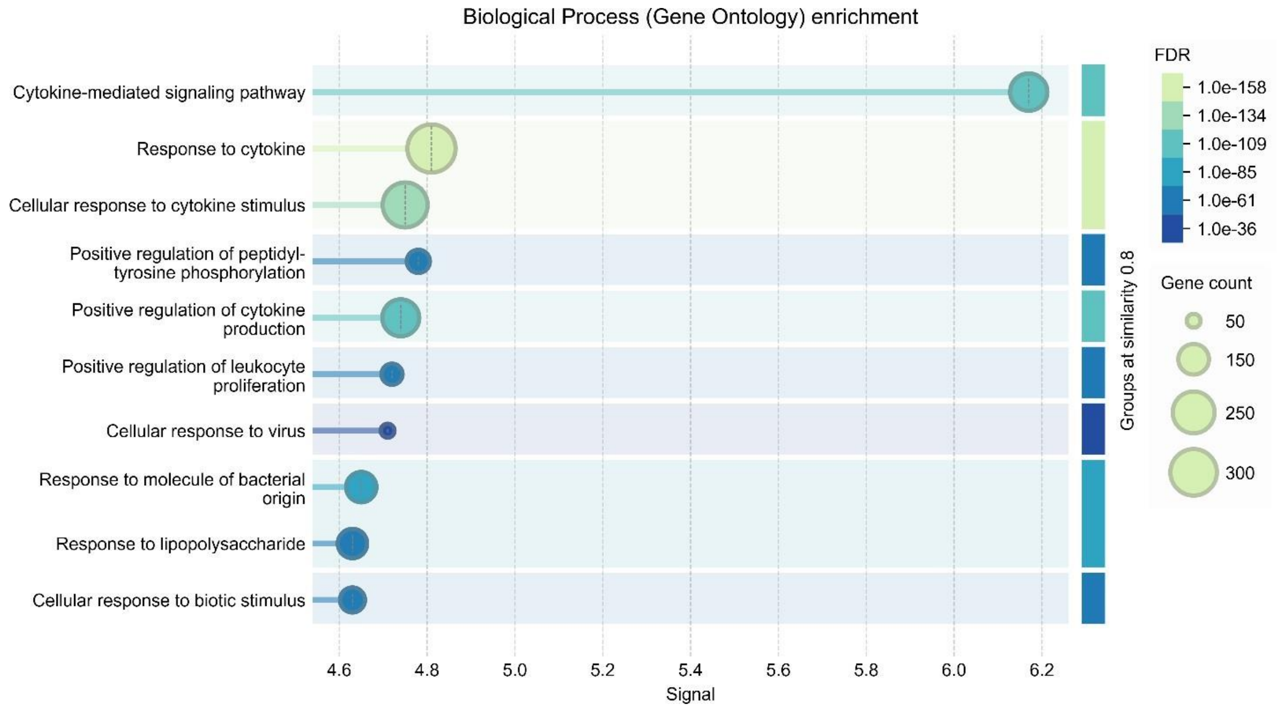

Figure S2 – The most significant biological GO Processes of the interactome in **Figure S1**.

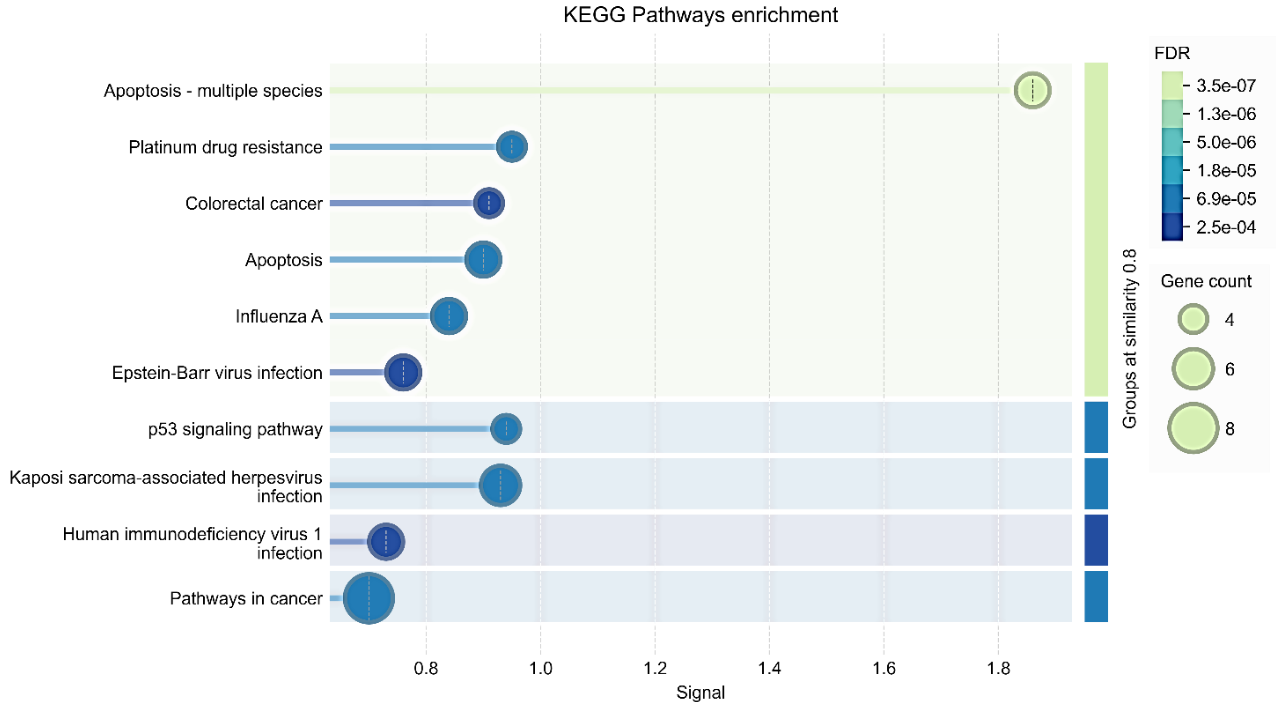

Figure S3 - The most significant KEGG Processes of the interactome in **Figure S1**.

Could unformylated MTRNR2L8/L12 interact with FPR2 under normal conditions?

This is a very important question because a negative answer would make it difficult to accept the complex functional model derived from the 1033-interactome. Harada M. et al. [1] demonstrated that N-formylated humanin activates both formyl peptide receptor-like 1 and 2 (FPRL1 or FPR2 and FPRL2), where FPRL1 (FPR2), known to be a promiscuous receptor, also binds a wide range of non-formylated peptides [2]. Some FPR receptor agonists are potential candidates as allosteric or ago-allosteric modulators of these receptors. In particular, FPR2 appears to have followed a different evolutionary path from FPR1, with a distinct recognition profile that includes binding to a wide range of much longer endogenous and microbial peptides [3] with 10^{-9} - 10^{-7} M range [3].

Harada et al., also found that unformylated humanin (HN) binds FPR2 with an EC50 of 0.012 nM, compared to 3.5 nM for fHN (Humanin) [1]. Our results show that FPR2 interacts significantly with both MTRNR2L8 and MTRNR2L12. Meanwhile, our interactomic model shows that FPR2 and the two mitochondrial peptides specifically interact with each other and with G proteins (GNAI2, GNB1, and GNG2) through highly reliable, experimentally validated interactions (confidence score between 0.800 and 0.999). GNAI2 is peculiar because the PDB entry 7wvx (2022) shows the cryo-EM structure of human FPR2 in complex with fHN and GNAI2 [4]. However, no additional data on the interaction between unformylated mitochondrial peptides and FPR2 or GNAI2 are available in the literature. A valid question arises: could unformylated MTRNR2L8/L12 interact with FPR2 under normal conditions? Several key factors must be considered.

Structural Aspects: Unformylated MTRNR2L8/L12 and Leucine vs. Serine - MTRNR2L8/L12 and HN share a 24-residue sequence [5,6], but they differ at residue 12, where HN-(L12) becomes MTRNR2L8/L12-(S12). Leucine is a neutral, hydrophobic, and bulky amino acid that favors hydrophobic interactions. Serine is neutral, polar, and small, and can form hydrogen bonds and minor hydrophobic interactions. The L12→S12 change results in the loss of a hydrophobic bulky interaction. The Cryo-EM structure (PDB: 7wvx), although it refers to the formylated form, shows an interaction in which the peptide Humanin and Aβ42 bind FPR2 via the N-terminal domain, fitting into a hydrophobic pocket formed by transmembrane helices III, V, VI, and VII. Binding is mainly driven by hydrophobic interactions and N-terminal interactions (formylated or not). This indicates that leucine is a key residue in alpha-helix/membrane or alpha-helix/protein pocket interactions due to its aliphatic side chain. Therefore, replacing L12 with S12 removes a hydrophobic contact point and increases polarity at that position. This likely reduces affinity (resulting in a higher Kd), since hydrophobic interaction is less effective at anchoring the MTRNR2L8/L12 peptide in the FPR2 pocket. The peptides are helical, and leucine stabilizes alpha-helices well. Serine has a lower tendency to form helices and can sometimes act as a structural "switch." If serine weakens or subtly modifies the helix in the central region, this could impair the correct presentation of the serine to the FPR2 binding site.

Affinity - The affinity of HN for FPR2 is 291-fold lower than that of the fNH form [3] but remains relatively high in a biological context (nanomolar range). Therefore, the interaction may still be biologically relevant under basal conditions, even if it is weaker than that of the formylated form, especially if the HN concentration in the cellular microenvironment is sufficient. Thus, regarding humanin, the hypothesis of an interaction and activity under normal (basal) conditions or in the absence of intense stress is well supported by the data.

The key now is to understand how the L12→S12 substitution can affect the affinity for the unformylated peptides, MTRNR2L8 and MTRNR2L12. The interaction under normal (basal) conditions depends on: 1) the physiological concentration of endogenous peptides at the receptor; 2) the extent of further decrease in affinity caused by the K12→S12 substitution in the MTRNR2L8/L12 peptides. Unfortunately, their affinities and concentrations are not reported in the literature, but the unformylated peptides likely interact with FPR2 under normal conditions, though with varying affinities. The three molecules are agonists, but they operate within different potency ranges. Formylation acts as a switch for maximum response; unformylated humanin is the potent basic agonist; and MTRNR2L8/L12 are weak, low-efficacy agonists of the base modulators and therefore not full agonists [6].

The L12→S12 substitution reduces affinity and probably also efficacy, but does not eliminate receptor recognition. The most critical structural data is always from the 7wvx complex. Its structure (FPR2–fHN–GNAI2) provides valuable insight for three key reasons: 1) The peptide penetrates deeply into the transmembrane pocket, ruling out a purely electrostatic or surface interaction. 2) The N-terminus mainly drives initial recognition, increasing affinity for formylation, but does not create the binding site from scratch. This aligns with the fact that unformylated HN should still bind FPR2 at concentrations near ~nM. 3) This arrangement is compatible with GNAI2 already being prefixed, meaning the system is predisposed to respond even to "weak" ligands. These considerations are essential because FPR2 is not simply an "on/off" receptor but a highly adaptable GPCR, capable of a wide range of efficacies. The L12→S12 substitution causes a real but manageable penalty. Leucine should contribute a hydrophobic enthalpic effect and stabilize helices. Serine results in a loss of hydrophobic packing, but it can form H-bonds with polar residues in the pocket

and shows greater local flexibility. Essentially, we face an enthalpic penalty but gain an entropic advantage. In a GPCR like FPR2, a slight loss of rigidity is not necessarily negative: it can promote promiscuous, low-affinity binding, which is precisely what a danger-sensing receptor needs.

From the geometry of the 7WVX complex, L12 does not appear to be a primary anchor residue. Instead, it is more likely a residue that stabilizes the helical structure. Therefore, a mutation from L12 to S12 shifts the conformational equilibrium but does not disrupt it. That the interactome reports: a) FPR2 ↔ MTRNR2L8/L12; b) FPR2 ↔ GNAI2; c) MTRNR2L8/L12 ↔ GNAI2 (directly or indirectly) is more than a detail. This suggests that FPR2 may exist in pre-formed complexes with GNAI2, indicating that the peptide does not need to "activate" the receptor from scratch but only to stabilize an already permissive conformation. In this scenario, even a weak agonist can produce a biologically relevant signal. This also aligns with basal signaling, anti-inflammatory modulation, and bias toward survival pathways (Gi/βγ, ERK, PI3K). Even without data on endogenous concentrations, we can infer that HN and MTRNR2Lx are constitutively expressed. They may be secreted locally, released in pericellular micro-domains, or concentrated near the receptor (paracrine/autocrine). In such a microenvironment, even a ligand with a Kd of 1.0×10^{-50} nM can be effective, especially if the receptor is highly expressed, preassembled, and not desensitized. In summary, as mentioned earlier, formylation is the key trigger for maximal response; unformylated humanin is the primary potent agonist; and MTRNR2L8/L12 are most likely the central weak agonists or modulators. As a result, the mitochondrial system regulates signal intensity to maintain homeostasis, preventing weak signals from being lost and strong ones from being detrimental. It enhances receptor activation without directly binding to the active site and boosts the response to weak stimuli. Thus, unformylated MTRNR2 L8/L12 could normally interact with FPR2 with high likelihood, although with lower affinity than unformylated HN, and likely with reduced efficacy and bias, functioning more as tonic modulators rather than triggers. If they did not interact under basal conditions, their functional conservation and co-emergence in the interactome with FPR2 and GNAI2 would be challenging to explain. Interaction analysis indicates that MTRNR2L8 and MTRNR2L12 directly interact with FPR2 and GNAI2 (confidence scores> 0.700). They are positioned at the periphery of an interactome with a multifunctional core and primarily serve regulatory roles within functional modules.

Table S2 - Mitochondrial gain control system

Ligand	Affinity (E50)	Effectiveness	Role
fHN	Maximum (0.012 nM)	Full agonist	Acute damage signal
HN	High (3.5 nM)	Partial agonist	Homeostasis/protection
MTRNR2L8/L12	Medium–low (nM-μM)	Weak agonists/PAM-like	Basal tone

Note: PAM stands for Positive Allosteric Modulator.

The parametric conditions used to generate this interactome are very robust, eliminating noise, database bias, or implausible signals. In well-structured biological networks, the multi-modular core is dominated by housekeeping, canonical transduction, and structurally or metabolically central proteins. The periphery typically contains sensors, regulators, contextual modulators, and endogenous ligands. That MTRNR2L8 and MTRNR2L12 are peripheral but have

direct connections to key nodes (FPR2, GNAI2) is precisely what one would expect from low-molecular-weight regulatory peptides. If they were in the core, it would be highly suspicious. Therefore, the FPR2–GNAI2 interaction exhibits specific features: FPR2 interacts promiscuously with GPCRs, integrates pro- and anti-inflammatory signals, and exhibits high conformational plasticity. GNAI2 acts as a downstream signaling hub that regulates chemotaxis, cAMP signaling, and ERK/PI3K signaling, and fine-tunes cellular responses. This axis does not form a module; it modulates them. Its role in managing regulatory activities across functional modules aligns well with the current understanding of its biology. From a topological interactomics viewpoint, a confidence score > 0.800 for a short peptide lacking canonical domains or large structural surfaces suggests the interaction is not random. This likely results from co-IPs, specific interactions, or the convergent recurrence of independent evidence. The network recognizes MTRNR2L8/L12 as fundamental functional components rather than noise.

From a network-theory perspective, nodes such as MTRNR2L8/L12, which have low degree centrality but high local betweenness, do not connect many nodes but instead influence how modules respond to stimuli. This is typical of endogenous ligands that fine-tune signals and systems that adapt to chronic stress. In simple terms, MTRNR2L8 and L12 do not activate the system but regulate its activity level. From the mitochondrion's perspective, it is not just a powerhouse; it is also a sensory and signaling organelle. This evidence indicates that the other MDPs (MOTS-c and SHLP) might not activate primary pathways but rather affect existing ones. The interactome revealed the functional periphery's role, its interactions with context-sensitive GPCRs, and its lack of structural centrality. MTRNR2L8 and MTRNR2L12 are not core structural nodes in the interactome but rather peripheral regulators that, via FPR2–G proteins, modulate the dynamic responses of key functional modules. Their role supports basal tonic activity and becomes more significant under sublethal mitochondrial stress. The findings also highlight an important point: the FPR2 system is not only a DAMP/PAMP receptor (damage-related molecular patterns/pathogen-associated molecular patterns) but also a receptor for mitochondrial "resilience" signals. By connecting innate immunity, mitochondrial homeostasis, and regulating inflammation, this challenges traditional views. What we observe is not a network artifact but an actual pattern of low-affinity, modulatory mitochondrial peptides acting on control, rather than structural, nodes. Therefore, we can hypothesize that MTRNR2L8 and MTRNR2L12 are endogenous FPR2 ligands that elicit Gi-biased, β–arrestin–low signaling with tonic homeostatic and anti-damage functions. This hypothesis is supported by a structural reference indicating that, in the FPR2–fHN–GNAI2 (7WVX) complex, fHN stabilizes transmembrane segments TM3–TM6–TM7, creating a conformation optimal for GNAI2 activation through a "classical GPCR Gi-like" mechanism, which results in cAMP inhibition, rapid ERK activation, and possible β-arrestin recruitment. Formylation promotes the "full agonist" conformation, thereby enabling internalization, desensitization, and robust inflammatory or chemotactic signals.

Our analysis shows that reducing affinity does not decrease the number of potential conformations. Binding, pocket closure, or 'fully active' conformation stabilization is likely unhindered by the L12→S12 substitution. The expected outcome is a reduction in states favoring β-arrestin and an increase in Gi-permissive but sub-active states. The presence of MTRNR2L8/L12 ↔ GNAI2 shows that FPR2 may be pre-coupled to GNAI2, that the peptide does not cause significant rearrangements, and that it only stabilizes a minimal intracellular opening. Here, Gi is activated, β-arrestin recruitment is inefficient, and receptor internalization does not happen. This mechanism is like that of biased μ-opioid receptor agonists and partial β-adrenergic receptor agonists.

Bibliography

1] Harada, M., Habata, Y., Hosoya, M., Nishi, K., Fujii, R., Kobayashi, M., & Hinuma, S. (2004). N-Formylated humanin activates both formyl peptide receptor-like 1 and 2. *Biochemical and biophysical research communications*, *324*(1), 255-261. https://doi.org/10.1016/j.bbrc.2004.09.046
2] Napolitano, F., & Montuori, N. (2025). The N-formyl peptide receptors: much more than chemoattractant receptors. Relevance in health and disease. *Frontiers in Immunology*, *16*, 1568629. https://doi.org/10.3389/fimmu.2025.1568629
3] He H-Q, Ye RD. The Formyl Peptide Receptors: Diversity of Ligands and Mechanism for Recognition. Molecules. 2017; 22(3):455. https://doi.org/10.3390/molecules22030455
4] Zhu, Y., Lin, X., Zong, X. et al. Structural basis of FPR2 in recognition of Aβ42 and neuroprotection by humanin. Nat Commun 13, 1775 (2022). https://doi.org/10.1038/s41467-022-29361-x
5] Hashimoto, Y.; Niikura, T.; Ito, Y.; Sudo, H.; Hata, M.; Arakawa, E.; Abe, Y.; Kita, Y.; Nishimoto, I. Detailed Characterization of Neuroprotection by a Rescue Factor Humanin against Various Alzheimer's Disease-Relevant Insults. *The Journal of Neuroscience* **2001**, *21*, 9235–9245, doi:10.1523/JNEUROSCI.21-23-09235.2001.
6] Niikura, T. Humanin and Alzheimer's Disease: The Beginning of a New Field. *Biochimica et Biophysica Acta (BBA) - General Subjects* **2022**, *1866*, 130024, doi:10.1016/j.bbagen.2021.130024.

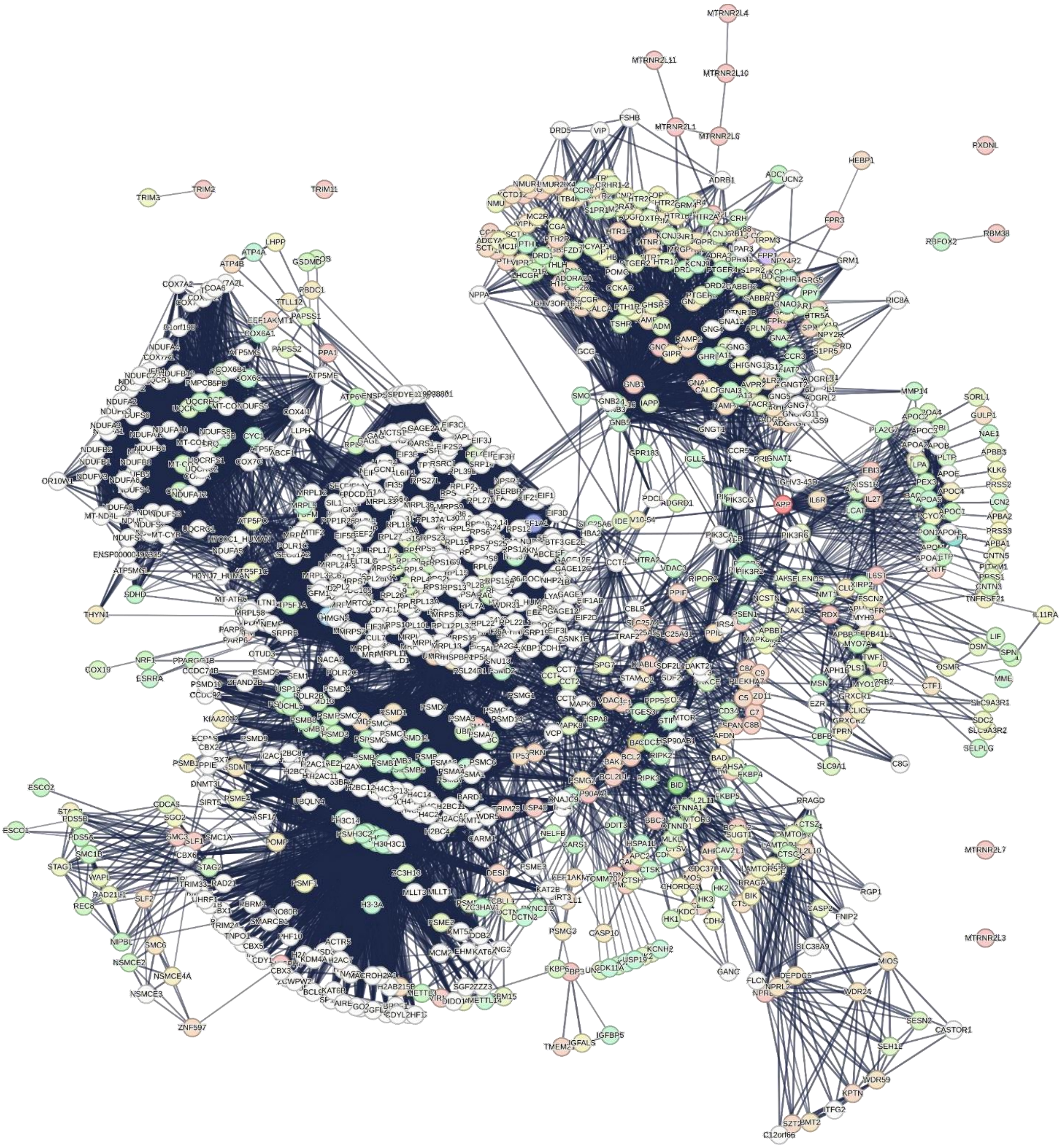

Figure S4 – Interactome-1041. The figure shows the same interactome as in **Figure S1**, but with a coefficient score of 0.700 and without the text-mining data. The topological parameters show: number of edges: 27,059; average node degree: 51.9; average local clustering: 0.758; expected number of edges: 11,831; PPi enrichment p-value: < 1.0e–16. Eight nodes are not connected to the central interactome. With this new setup, the interactome exhibits well-defined functional modules (subgraphs), and humanins are consistently at the periphery of the system.

Table S3 – Enrichment of functional terms of Interactome_1041 with conf.score 0.700 and no Text-mining.

Biological Process (Gene Ontology)	download	742 GO-terms significantly enriched; file-format: tab-delimited
Molecular Function (Gene Ontology)	download	121 GO-terms significantly enriched; file-format: tab-delimited
Cellular Component (Gene Ontology)	download	197 GO-terms significantly enriched; file-format: tab-delimited
Reference Publications (PubMed)	download	10000 publications significantly enriched; file-format: tab-delimited
Local Network Cluster (STRING)	download	209 clusters significantly enriched; file-format: tab-delimited
KEGG Pathways	download	126 pathways significantly enriched; file-format: tab-delimited
Reactome Pathways	download	418 pathways significantly enriched; file-format: tab-delimited
WikiPathways	download	123 pathways significantly enriched; file-format: tab-delimited
Disease-gene Associations (DISEASES)	download	17 diseases significantly enriched; file-format: tab-delimited
Tissue Expression (TISSUES)	download	119 tissues significantly enriched; file-format: tab-delimited
Subcellular Localization (COMPARTMENTS)	download	183 compartments significantly enriched; file-format: tab-delimited
Human Phenotype (Monarch)	download	191 phenotypes significantly enriched; file-format: tab-delimited
Annotated Keywords (UniProt)	download	65 keywords significantly enriched; file-format: tab-delimited
Protein Domains (Pfam)	download	11 domains significantly enriched; file-format: tab-delimited
Protein Domains and Features (InterPro)	download	87 domains significantly enriched; file-format: tab-delimited
Protein Domains (SMART)	download	23 domains significantly enriched; file-format: tab-delimited
**All enriched terms** (without PubMed)	download	2642 enriched terms in 15 categories; file-format: tab-delimited
Selected terms only	download	no enriched terms selected (click on any term above to select)

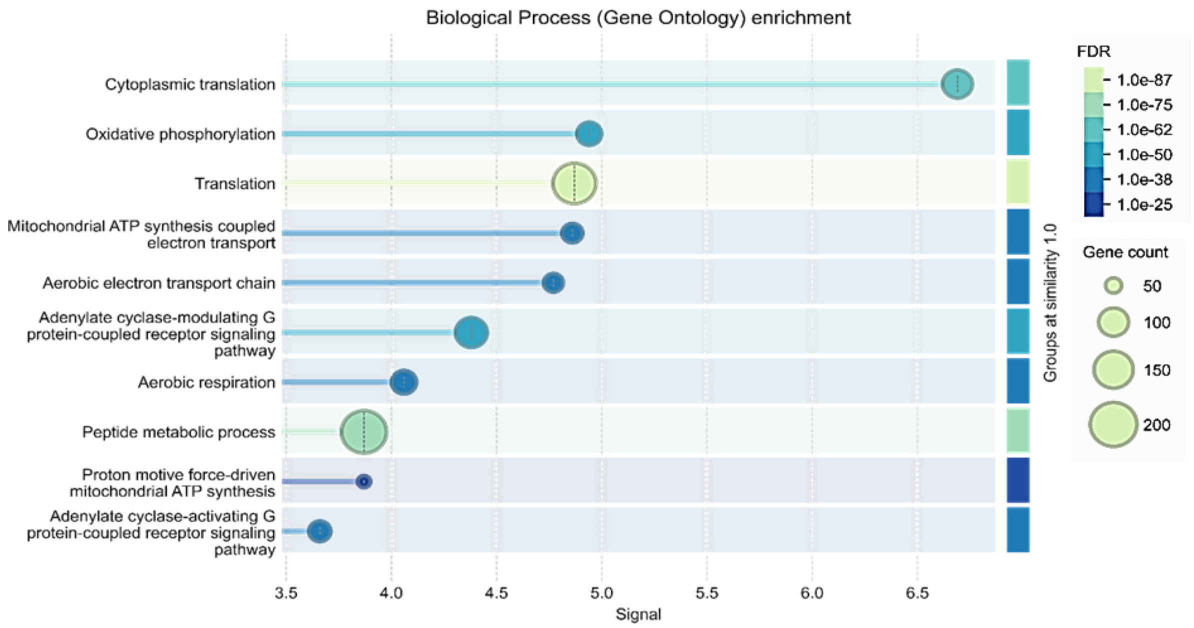

Figure S5 - The most significant biological GO Processes of the interactome-1041.

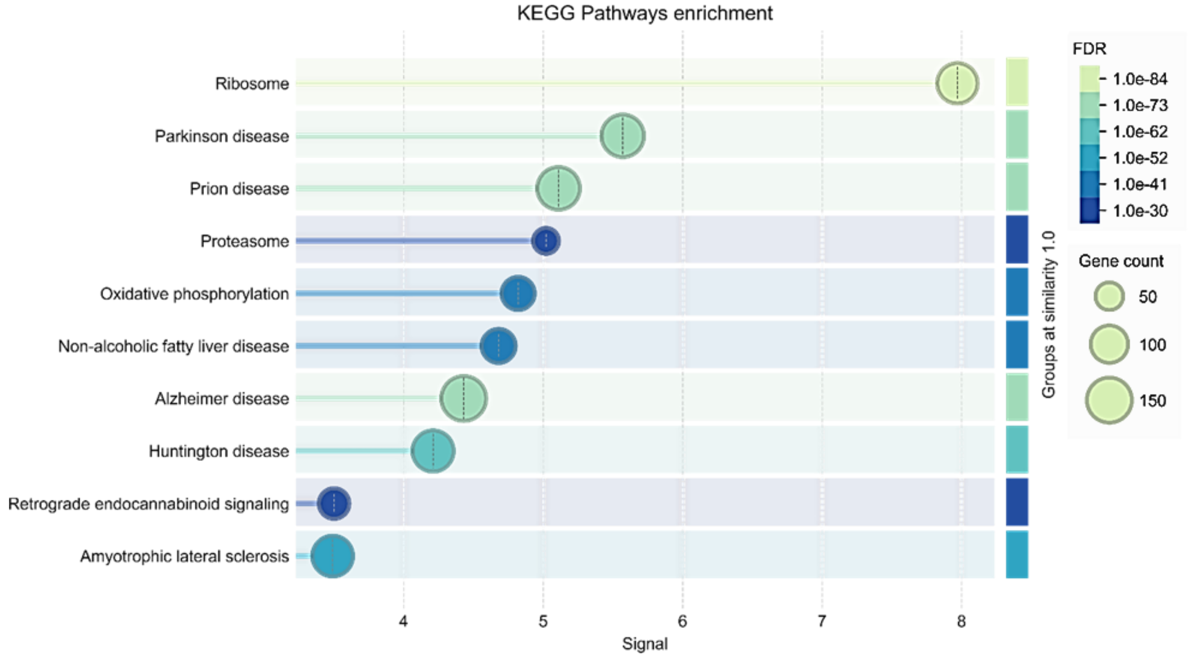

Figure S6 - The most significant KEGG Processes of interactome-1041.

Table S4 – Enrichment of functional terms of hub-and-spoke with conf. score 0.700.

(The sets were filtered according to the display settings)

Biological Process (Gene Ontology)	download	*151 GO-terms significantly enriched; file-format: tab-delimited*
Molecular Function (Gene Ontology)	download	*31 GO-terms significantly enriched; file-format: tab-delimited*
Cellular Component (Gene Ontology)	download	*63 GO-terms significantly enriched; file-format: tab-delimited*
Reference Publications (PubMed)	download	*21 publications significantly enriched; file-format: tab-delimited*
Local Network Cluster (STRING)	download	*30 clusters significantly enriched; file-format: tab-delimited*
KEGG Pathways	download	*80 pathways significantly enriched; file-format: tab-delimited*
Reactome Pathways	download	*156 pathways significantly enriched; file-format: tab-delimited*
WikiPathways	download	*75 pathways significantly enriched; file-format: tab-delimited*
Disease-gene Associations (DISEASES)	download	*5 diseases significantly enriched; file-format: tab-delimited*
Tissue Expression (TISSUES)	download	*95 tissues significantly enriched; file-format: tab-delimited*
Subcellular Localization (COMPARTMENTS)	download	*63 compartments significantly enriched; file-format: tab-delimited*
Human Phenotype (Monarch)	download	*136 phenotypes significantly enriched; file-format: tab-delimited*
Annotated Keywords (UniProt)	download	*15 keywords significantly enriched; file-format: tab-delimited*
Protein Domains (Pfam)	download	*2 domains significantly enriched; file-format: tab-delimited*
Protein Domains and Features (InterPro)	download	*16 domains significantly enriched; file-format: tab-delimited*
Protein Domains (SMART)	download	*6 domains significantly enriched; file-format: tab-delimited*
**All enriched terms** (without PubMed)	download	*924 enriched terms in 15 categories; file-format: tab-delimited*
Selected terms only	download	*no enriched terms selected (click on any term above to select)*

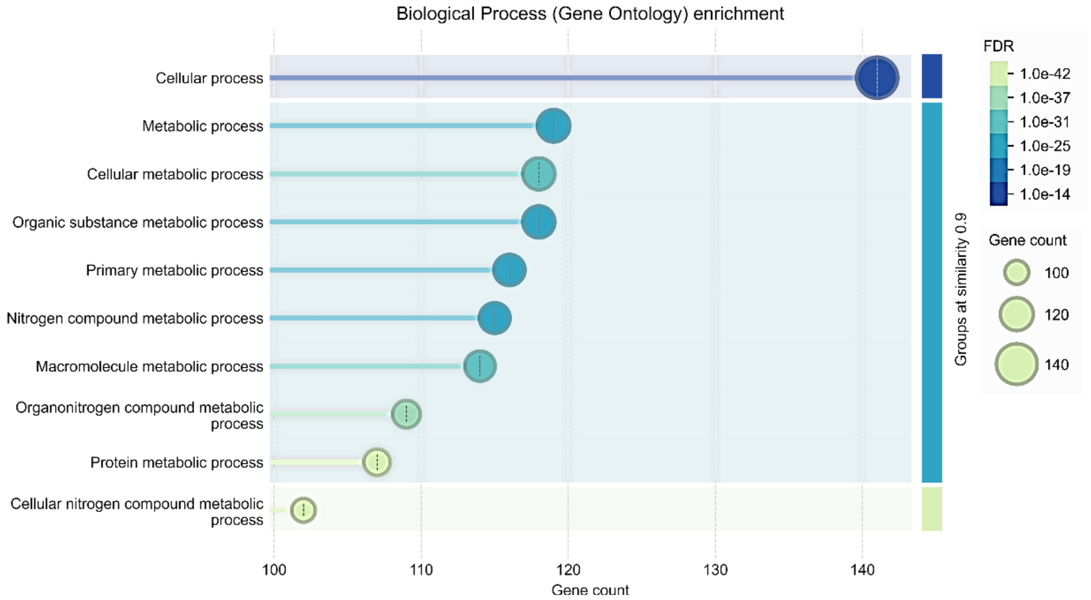

Figure S7 – Gene counts for the most significant Biological Process (Gene Ontology) terms enriched in the hub-and-spoke model. Bubble size indicates gene count, and color indicates FDR.

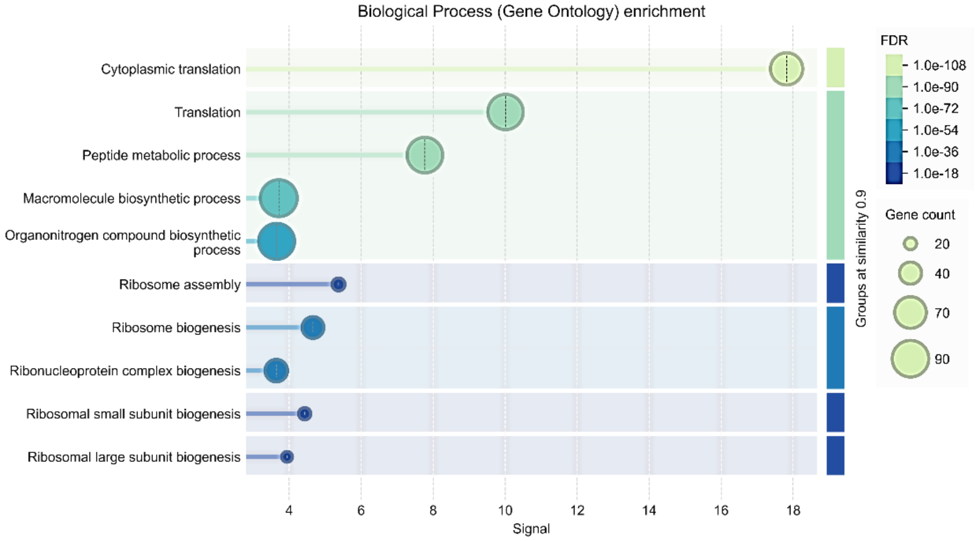

Figure S8 – Signal values for the most significant Biological Process (Gene Ontology) terms enriched in the hub-and-spoke model. Bubble size indicates gene count, and color indicates FDR.

Table S5 – Main biological process driven by hub-and-spoke model.

Biological Process (GO)					
GO-term	Description	Count in network	Strength	Signal	FDR
GO:0002181	Cytoplasmic translation	73 of 123	1.92	17.82	3.27e-108
GO:0006412	Translation	85 of 389	1.48	10.01	3.33e-98
GO:0006518	Peptide metabolic process	86 of 547	1.34	7.77	7.83e-89
GO:0042255	Ribosome assembly	21 of 60	1.69	5.37	1.10e-24
GO:0042254	Ribosome biogenesis	39 of 299	1.26	4.66	2.66e-33
GO:0022613	Ribonucleoprotein complex biogenesis	41 of 449	1.1	3.64	1.48e-29
GO:0010467	Gene expression	93 of 2101	0.79	2.6	4.24e-51
GO:1901796	Regulation of signal transduction by p53 class mediator	13 of 105	1.23	1.99	9.89e-10
GO:1902253	Regulation of intrinsic apoptotic signaling pathway by p53 class media...	8 of 32	1.54	1.78	1.17e-07
GO:0006334	Nucleosome assembly	12 of 124	1.13	1.56	8.95e-08
GO:2001242	Regulation of intrinsic apoptotic signaling pathway	13 of 169	1.03	1.42	2.06e-07
GO:0031497	Chromatin assembly	13 of 179	1.0	1.35	3.91e-07
GO:0043933	Protein-containing complex organization	43 of 1465	0.61	1.31	4.89e-13
GO:0032200	Telomere organization	11 of 129	1.07	1.31	1.50e-06
GO:0044237	Cellular metabolic process	118 of 6568	0.4	1.12	4.17e-31
GO:0032543	Mitochondrial translation	9 of 112	1.05	1.01	4.97e-05

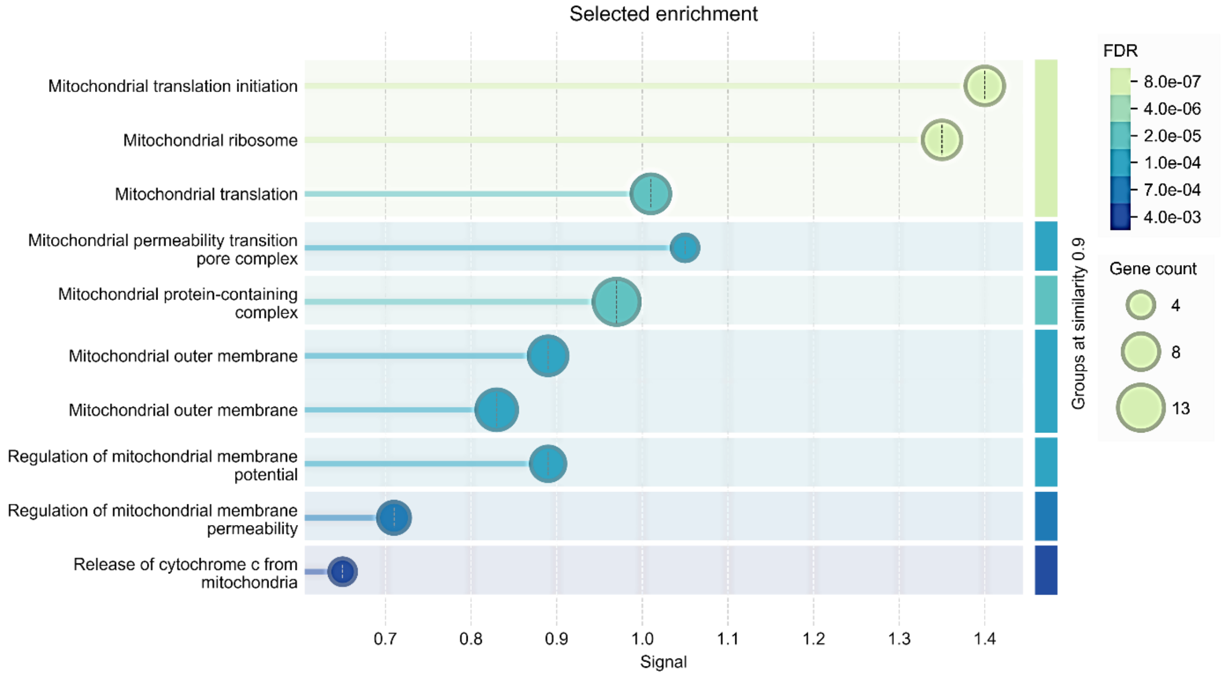

Figure S9 - Signal values of selected mitochondria-related enriched terms in the hub-and-spoke model. The plot highlights mitochondrial translation, mitochondrial ribosome, mitochondrial protein-containing complexes, mitochondrial membrane-related terms, regulation of mitochondrial membrane potential/permeability, and cytochrome c release.

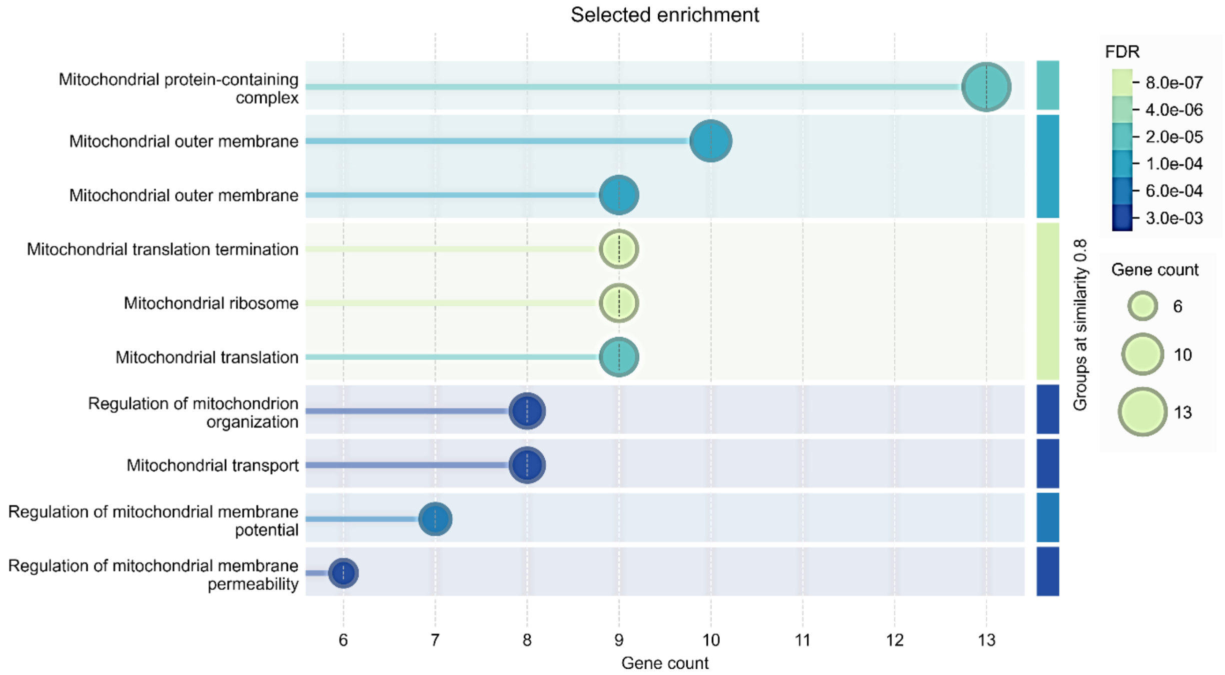

Figure S10 – Gene counts of selected mitochondria-related enriched terms in the hub-and-spoke model. Bubble size indicates gene count, and color indicates FDR.

Table S6 - Enrichment of functional terms of cluster 1 (conf.score 0.700).

Biological Process (Gene Ontology)	download	*319 GO-terms significantly enriched; file-format: tab-delimited*
Molecular Function (Gene Ontology)	download	*79 GO-terms significantly enriched; file-format: tab-delimited*
Cellular Component (Gene Ontology)	download	*133 GO-terms significantly enriched; file-format: tab-delimited*
Reference Publications (PubMed)	download	*10000 publications significantly enriched; file-format: tab-delimited*
Local Network Cluster (STRING)	download	*136 clusters significantly enriched; file-format: tab-delimited*
KEGG Pathways	download	*93 pathways significantly enriched; file-format: tab-delimited*
Reactome Pathways	download	*366 pathways significantly enriched; file-format: tab-delimited*
WikiPathways	download	*74 pathways significantly enriched; file-format: tab-delimited*
Disease-gene Associations (DISEASES)	download	*14 diseases significantly enriched; file-format: tab-delimited*
Tissue Expression (TISSUES)	download	*115 tissues significantly enriched; file-format: tab-delimited*
Subcellular Localization (COMPARTMENTS)	download	*138 compartments significantly enriched; file-format: tab-delimited*
Human Phenotype (Monarch)	download	*259 phenotypes significantly enriched; file-format: tab-delimited*
Annotated Keywords (UniProt)	download	*46 keywords significantly enriched; file-format: tab-delimited*
Protein Domains (Pfam)	download	*9 domains significantly enriched; file-format: tab-delimited*
Protein Domains and Features (InterPro)	download	*48 domains significantly enriched; file-format: tab-delimited*
Protein Domains (SMART)	download	*15 domains significantly enriched; file-format: tab-delimited*
**All enriched terms** (without PubMed)	download	*1844 enriched terms in 15 categories; file-format: tab-delimited*
Selected terms only	download	*no enriched terms selected (click on any term above to select)*

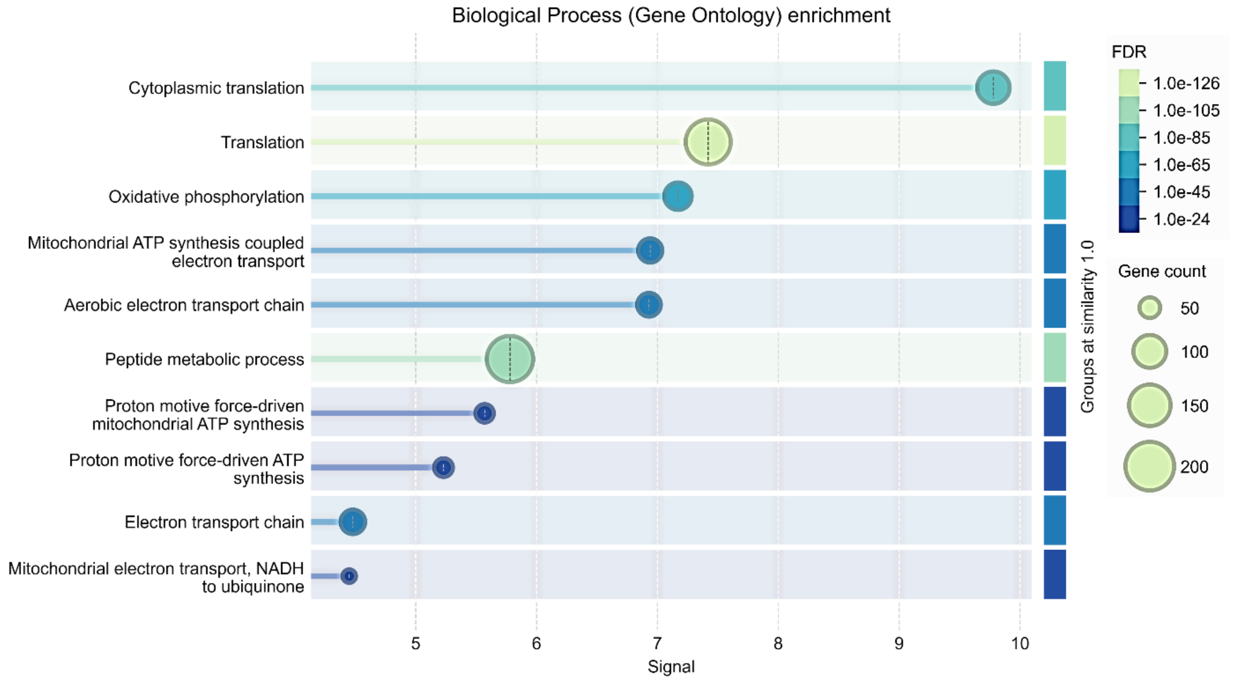

Figure S11 – Signal values of biological processes of cluster 1.

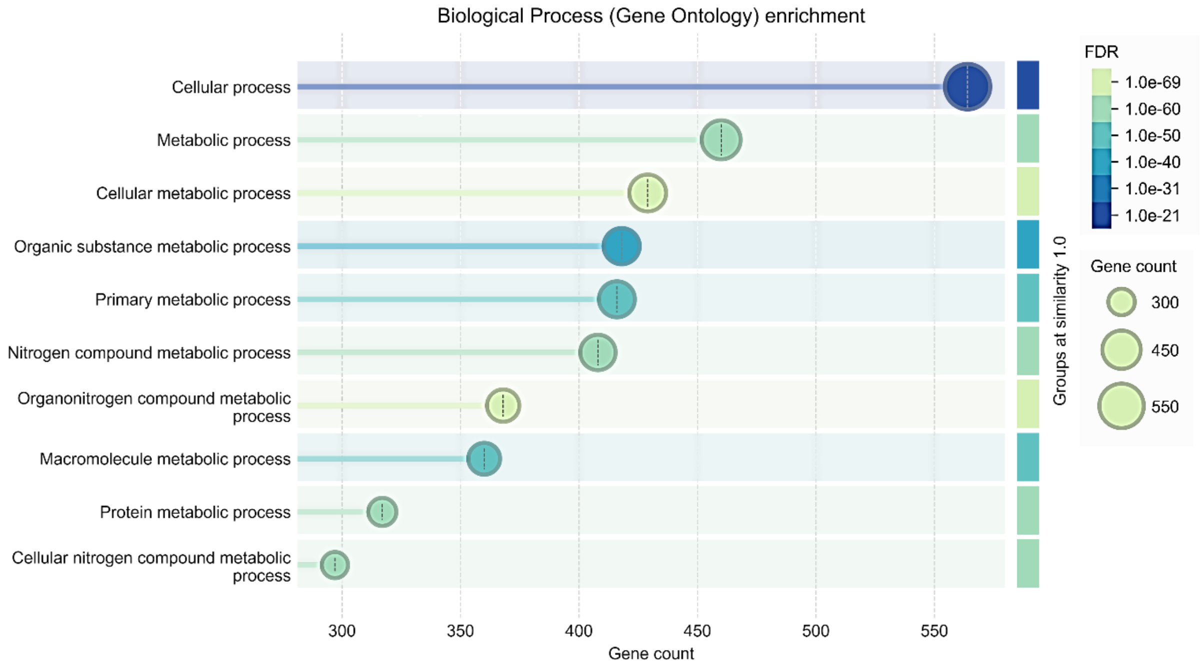

Figure S12 – Count of genes of biological processes in cluster 1.

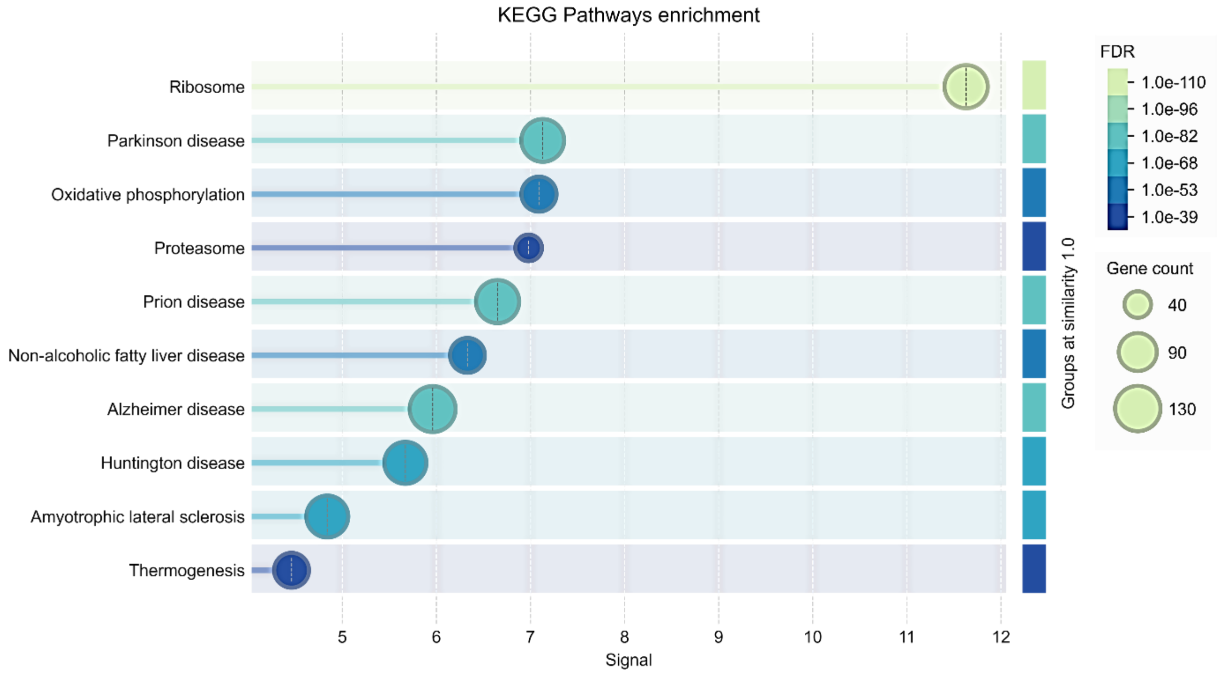

Figure S13 – Signal values in KEGG pathways of cluster 1.

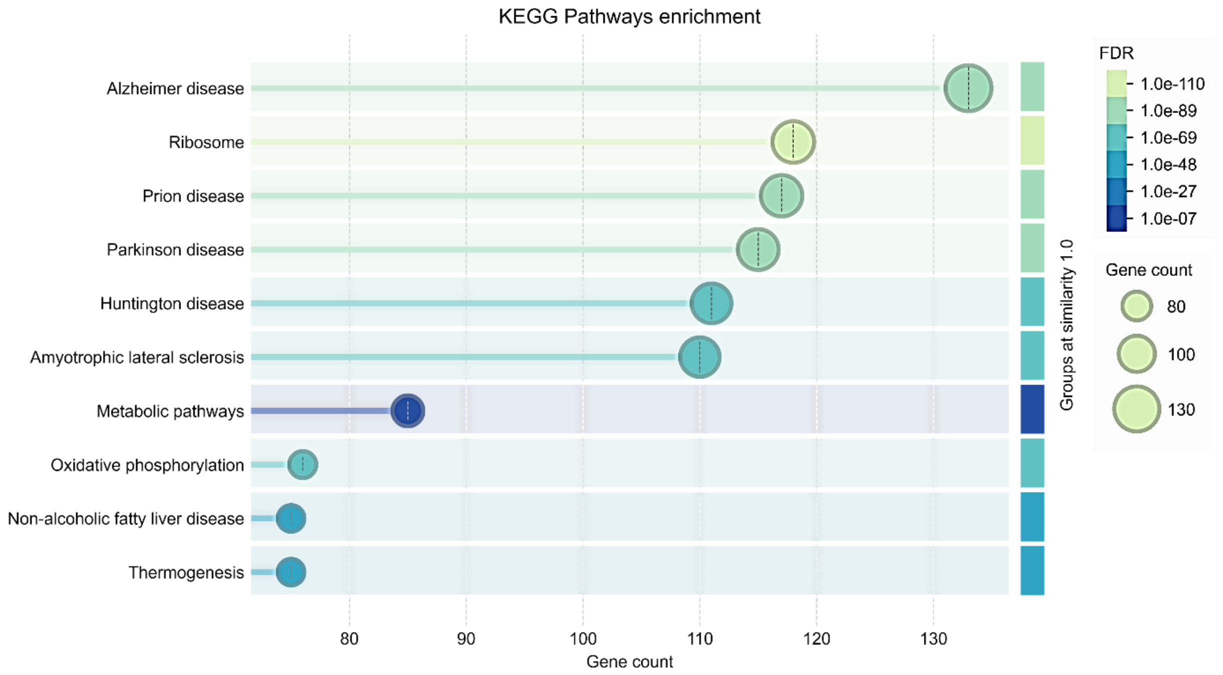

Figure S14 – Gene counts in KEGG pathways of cluster 1.

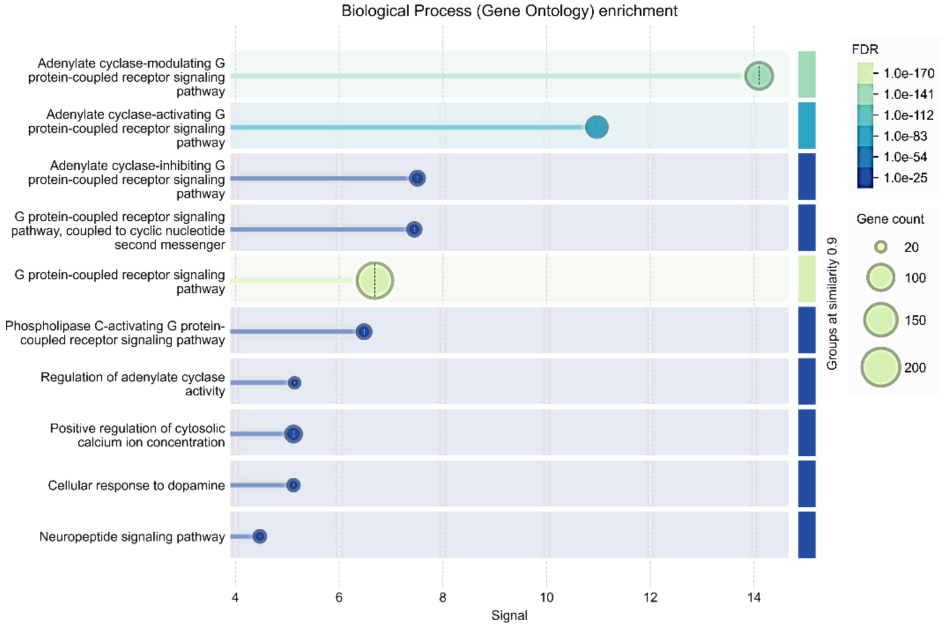

Figure S15 - Significant functional activities of Cluster 2.
